# The future directions of CAR-T Cell therapy: unlocking the potential of immunotherapy in cancer treatment

**DOI:** 10.3389/fmmed.2026.1931159

**Published:** 2026-09-15

**Authors:** Angel Alberto Justiz-Vaillant, Sachin Soodeen, Rodolfo Arozarena-Fundora, Patrick Eberechi Akpaka, Chandrashekhar Unakal

**Affiliations:** 1 Department of Pathology/Microbiology and Pharmacology, University of the West Indies, St. Augustine, Trinidad and Tobago; 2 North Central Regional Health Authority (NCRHA), Trinidad and Tobago, Trinidad and Tobago; 3 Department of Clinical and Surgical Sciences, Faculty of Medical Sciences, The University of the West Indies, St. Augustine, Trinidad and Tobago

**Keywords:** allogeneic CAR-T cells, CAR cost, CAR-T cell therapy, cytokine release syndrome, multi-target CAR-T cells, precision medicine, predictive biomarkers, solid tumours

## Abstract

Chimeric antigen receptor (CAR) T-cell therapy has transformed the treatment landscape for relapsed and refractory haematological malignancies, producing durable clinical responses in patients with otherwise limited therapeutic options. Despite these advances, significant biological, clinical, and economic barriers continue to limit its broader application, particularly in solid tumours. This literature review critically evaluates recent developments in CAR-T cell therapy, with emphasis on areas that have received limited discussion in previous reviews. Multi-target CAR strategies, including bispecific, tandem, and pooled CAR-T cell approaches, are comparatively assessed with respect to antigen escape, manufacturing complexity, therapeutic efficacy, and safety. Current challenges in solid tumour treatment are examined through evidence from clinical trials, highlighting key obstacles such as antigen heterogeneity, the immunosuppressive tumour microenvironment, poor cellular trafficking, and treatment-related failures that have informed the development of next-generation CAR constructs. Emerging approaches to overcoming antigen loss, including γδ CAR-T cells, are reviewed alongside their current limitations, including restricted *ex vivo* expansion, donor variability, isolation challenges, and uncertain long-term persistence. The review also addresses the growing importance of improving affordability and accessibility through allogeneic “off-the-shelf” CAR-T products, genome editing technologies, automated manufacturing platforms, and rational combination therapies designed to enhance efficacy while reducing treatment costs. In addition, advances in toxicity prediction are evaluated, focusing on biomarkers beyond interleukin-6, including ferritin, C-reactive protein, soluble interleukin-2 receptor alpha, and multiparametric cytokine signatures for the early identification of severe cytokine release syndrome. Collectively, these developments demonstrate that future progress in CAR-T cell therapy will depend not only on enhancing antitumour efficacy but also on improving safety, accessibility, manufacturing efficiency, and equitable global implementation through evidence-based innovation.

## 1 CAR-T-cell therapy: clinical progress, limitations, and future directions

CAR-T-cell therapy has transformed the treatment of selected relapsed or refractory haematological malignancies, particularly B-cell acute lymphoblastic leukaemia, non-Hodgkin lymphoma, and multiple myeloma (Zhang et al., 2022; Schuster et al., 2019; Sang et al., 2020). Multiple autologous CD19^−^and BCMA-directed CAR-T-cell products have received FDA approval for relapsed or refractory B-cell malignancies and multiple myeloma, including tisagenlecleucel, axicabtagene ciloleucel, lisocabtagene maraleucel, idecabtagene vicleucel, and ciltacabtagene autoleucel (Neelapu et al., 2017; Jacobson et al., 2022; Locke et al., 2019; Watanabe et al., 2022; Labbé et al., 2021; Kamdar et al., 2022; Sehgal et al., 2022; Kharfan-Dabaja et al., 2022; Munshi et al., 2021; Hansen et al., 2023; Jagannath et al., 2021; Berdeja et al., 2021; Mi et al., 2023). Nevertheless, therapeutic success remains concentrated in B-cell malignancies, and relapse, manufacturing complexity, cost, treatment-related toxicity, and limited accessibility continue to restrict broader clinical implementation.

Current development is increasingly focused on multi-antigen targeting to reduce tumour escape. Dual, tandem, or sequential CAR strategies may improve recognition of heterogeneous tumour populations and decrease the likelihood that loss of a single antigen will result in relapse (Lindo et al., 2020; Bell and Gottschalk, 2021). However, broader targeting can increase receptor complexity, alter signalling strength, and potentially increase on-target/off-tumour toxicity. Improving CAR-T-cell persistence is therefore equally important. Optimisation of costimulatory domains, metabolic fitness, cytokine signalling, and memory-cell differentiation may prolong antitumour activity, although excessive stimulation can also accelerate exhaustion or increase inflammatory toxicity (López-Cantillo et al., 2022).

Translation into solid tumours remains considerably less successful. Major barriers include heterogeneous antigen expression, poor trafficking, dense stromal architecture, limited persistence, and an immunosuppressive tumour microenvironment containing regulatory immune cells, inhibitory cytokines, and checkpoint pathways (Corti et al., 2022; Guzman et al., 2023). Armoured CARs, regional delivery, checkpoint inhibition, and multi-target constructs are being investigated to overcome these barriers, but most evidence remains preclinical or derived from small early-phase trials. Consequently, improved biological activity has not yet consistently translated into durable clinical benefit in solid cancers.

Safety also remains a major determinant of treatment feasibility. Cytokine release syndrome and immune effector cell-associated neurotoxicity syndrome can be life-threatening and require specialised monitoring and rapid intervention (Corti et al., 2022; Guzman et al., 2023). Although supportive management has improved, future progress will require predictive biomarkers, toxicity-adapted CAR designs, controllable activation systems, and dosing strategies that reduce pathological inflammation without compromising antitumour efficacy.

Target-antigen biology is central to both efficacy and resistance. CD19 remains the most clinically validated target because of its broad expression across B-cell malignancies and its limited distribution outside the B-cell lineage (Roex et al., 2022; Viardot et al., 2020; Hay and Turtle, 2017). However, CD19 downregulation or loss can produce antigen-negative relapse, while inadequate CAR-T-cell persistence and an unfavourable tumour microenvironment may cause treatment failure even when CD19 expression is retained (Strati and Neelapu, 2021; Mulgaonkar et al., 2023). Alternative or complementary targets, including CD20 and CD22, have therefore been investigated through sequential, dual-target, and combinatorial approaches (Duell et al., 2024; Al-Haideri et al., 2022; Liu Z. et al., 2024; Baird et al., 2021). These strategies have produced encouraging responses, particularly after CD19-directed treatment failure, but they do not completely eliminate antigen escape and may select for increasingly complex patterns of tumour evolution.

Overall, CAR-T-cell therapy represents a major advance rather than a universally effective cancer treatment. Future success will depend on rational antigen selection, improved persistence, safer multi-target designs, more efficient manufacturing, and strategies capable of overcoming the biological complexity of solid tumours and treatment-resistant haematological malignancies.

## 2 Multi-target CAR T-Cell therapy

Multi-target chimeric antigen receptor (CAR) T-cell therapy has emerged as an important strategy for addressing tumour heterogeneity and antigen-negative relapse. Although single-target CAR T-cell products can induce deep and durable responses, particularly in B-cell malignancies, selective therapeutic pressure may favour the survival of tumour cells that have lost, downregulated, mutated, or otherwise concealed the targeted antigen. Simultaneous or sequential recognition of more than one tumour-associated antigen may therefore reduce, although not completely eliminate, antigen-mediated immune escape (Cordoba et al., 2021; Gómez-Melero et al., 2025; Xie et al., 2022; de Oliveira Canedo et al., 2025).

The term *multi-target CAR T-cell therapy* encompasses several distinct approaches, including pooled or sequential monospecific products, co-transduced products, bicistronic or parallel dual-CAR T cells, tandem CARs and more complex logic-gated systems (Xie et al., 2022; de Oliveira Canedo et al., 2025; Wang et al., 2025). These approaches should not be considered interchangeable because antigen recognition occurs differently in each design and their manufacturing, signalling and pharmacological characteristics vary substantially.

In pooled CAR T-cell therapy, two independently manufactured monospecific CAR T-cell populations are administered either together or sequentially. For example, separate CD19^−^and CD22-directed products may be used to broaden recognition of malignant B cells (Xie et al., 2022; de Oliveira Canedo et al., 2025). This modular strategy allows each CAR to be designed, manufactured and quality-controlled independently. It may also permit separate dose adjustment of the two products. However, individual T cells usually recognise only one antigen. Differences in expansion, trafficking, persistence or susceptibility to exhaustion can cause one population to dominate after infusion, reducing effective dual-target coverage. Producing two cellular products may also increase cost, release-testing requirements and logistical complexity (Xie et al., 2022; de Oliveira Canedo et al., 2025; Wang et al., 2025).

Bicistronic or parallel dual-CAR T cells contain two independent CARs within the same T cell, commonly encoded by a single vector. Each CAR possesses its own antigen-binding domain and signalling architecture, creating an “OR-gate” response in which recognition of either antigen can activate cytotoxicity (Xie et al., 2022; Wang et al., 2025). Because successfully engineered cells carry both specificities, this approach provides more consistent cell-by-cell antigen coverage than pooled products. Clinical studies of CD19/CD22-directed bicistronic CAR T cells have demonstrated feasibility and antitumour activity in B-cell acute lymphoblastic leukaemia and large B-cell lymphoma (Spiegel et al., 2021; Roddie et al., 2023). Nevertheless, dual-receptor constructs increase genetic payload size and may complicate vector production, transduction and balanced receptor expression. Signalling intensity is also design-dependent: poorly optimised receptors may promote tonic signalling or dysfunction, whereas appropriately balanced dual stimulation may support expansion and persistence. Therefore, exhaustion should not be regarded as an inevitable consequence of dual-CAR expression (Xie et al., 2022; Wang et al., 2025).

Tandem CARs, or TanCARs, incorporate two antigen-binding domains into a single receptor chain. Binding of either antigen can activate the T cell, while simultaneous engagement may increase functional avidity and stabilise the immune synapse (Gómez-Melero et al., 2025; Wang et al., 2025). Tandem designs ensure that both binding domains are present on every CAR-positive cell and generally require only one intracellular signalling module. CD19/CD20, CD19/CD22 and other tandem combinations have shown encouraging preclinical and early clinical activity (Gómez-Melero et al., 2025; Wang et al., 2025). However, tandem receptor performance is particularly sensitive to structural configuration. The order of the binding domains, linker flexibility, hinge length, epitope location, transmembrane region and affinity of each binder can influence antigen accessibility and signal transmission. A tandem construct may therefore perform strongly against dual-antigen-positive tumour cells yet recognise single-antigen-positive cells less efficiently when steric hindrance or suboptimal geometry occurs (Gómez-Melero et al., 2025; Wang et al., 2025).

Multi-targeting remains especially attractive for solid tumours, where antigen expression is frequently heterogeneous. Nevertheless, antigen multiplicity alone cannot overcome inadequate trafficking, dense stromal barriers, immunosuppressive cytokines, metabolic restriction, T-cell exhaustion or on-target/off-tumour toxicity (Chen T. et al., 2024). Moreover, tumours may escape by losing both targeted antigens or through antigen-independent resistance mechanisms.

Overall, multi-target CAR T-cell therapy broadens tumour recognition and can reduce vulnerability to single-antigen loss. Tandem CARs offer an integrated receptor but require extensive structural optimisation; bicistronic dual-CARs provide flexible same-cell recognition but increase genetic and signalling complexity; and pooled products preserve validated monospecific designs but provide less uniform cell-level coverage. Current evidence is promising, but it does not establish universal superiority over optimised single-target CAR T-cell therapy. Selection should therefore be guided by antigen density, co-expression patterns, disease biology, construct fitness, manufacturing feasibility and the mechanisms most likely to drive relapse (Cordoba et al., 2021; Gómez-Melero et al., 2025; Xie et al., 2022; de Oliveira Canedo et al., 2025; Wang et al., 2025; Spiegel et al., 2021; Roddie et al., 2023; Chen T. et al., 2024). Figure 1 shows critical comparison of multi-target CAR-T cell strategies.

**FIGURE 1:**
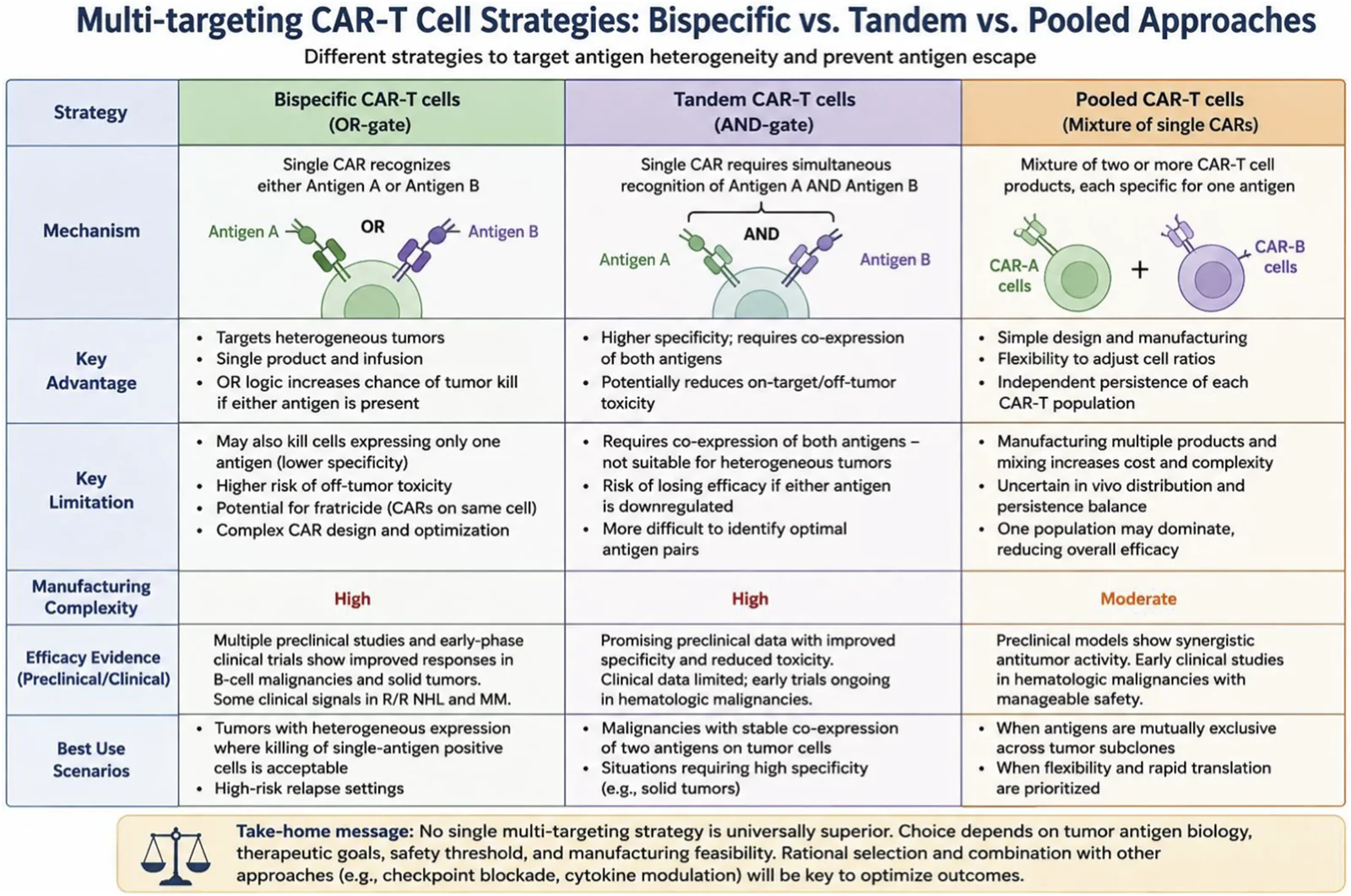
Critical comparison of multi-target CAR-T cell strategies—bispecific, tandem, and pooled CAR approaches—highlighting their mechanisms of action, advantages, limitations, manufacturing complexity, current clinical evidence, and potential applications for overcoming tumour antigen heterogeneity and antigen escape.

## 3 Generations of CAR-T cells

The evolution of CAR-T-cell therapy has been driven by the need to improve T-cell activation, persistence, and antitumour efficacy while reducing treatment failure and toxicity. Each successive CAR generation has sought to overcome the biological limitations of its predecessor; however, increased receptor complexity has not always translated into superior clinical outcomes.

First-generation CARs consisted of a single-chain variable fragment (scFv) linked to the CD3ζ signalling domain, providing the primary activation signal for T cells. Although these constructs demonstrated antigen-specific cytotoxicity, the absence of costimulatory signalling resulted in poor *in vivo* persistence, limited proliferation, and suboptimal clinical efficacy (Heuser et al., 2003).

Second-generation CARs incorporated a single costimulatory domain, such as CD28, 4-1BB, ICOS, CD134 (OX40), or CD27, markedly improving T-cell expansion, persistence, cytokine production, and resistance to exhaustion. These advances established second-generation CARs as the current clinical standard and formed the basis of most approved CAR-T-cell therapies (Guedan et al., 2018; Finney et al., 2004; Song et al., 2012).

Third-generation CARs combine two costimulatory domains to further enhance activation and long-term persistence. While preclinical studies have demonstrated improved T-cell survival and antitumour activity, clinical evidence has not consistently shown superiority over second-generation CARs, and stronger signalling may increase the risk of excessive immune activation and treatment-related toxicities (Guercio et al., 2021; Wei et al., 2022).

Fourth-generation CARs (“armoured” CAR-T cells) build upon second-generation constructs by incorporating inducible transgenes encoding immunomodulatory molecules, including IL-12 and IL-18. These cytokines remodel the tumour microenvironment, recruit endogenous immune cells, and enhance CAR-T-cell persistence, making this strategy particularly attractive for solid tumours (Luo et al., 2022; Avanzi et al., 2018; Chmielewski and Abken, 2017). Nevertheless, cytokine-mediated toxicities and the challenge of controlling transgene expression remain important barriers to clinical translation.

Overall, CAR evolution reflects a progressive refinement of T-cell engineering rather than a linear improvement in therapeutic efficacy. Future CAR designs are expected to integrate controllable signalling, genome editing, and synthetic biology approaches to improve safety, persistence, and activity against heterogeneous solid tumours.

## 4 Toxicities in CAR-T cell therapy

Although CAR-T-cell therapy has revolutionised the treatment of relapsed and refractory haematological malignancies, treatment-related toxicities remain a major limitation. These complications result from excessive immune activation, prolonged immune dysfunction, and, in allogeneic products, donor–recipient immune interactions.

Cytokine release syndrome (CRS) is the most frequent acute toxicity, driven by excessive production of inflammatory cytokines, particularly IL-1 and IL-6 (Obstfeld et al., 2017; Dong et al., 2021; Norelli et al., 2018). Management primarily involves tocilizumab, with corticosteroids reserved for severe cases (Gardner et al., 2019; Giavridis et al., 2018; Gazeau et al., 2023). However, reliable biomarkers predicting CRS severity remain unavailable.

Prolonged T- and B-cell aplasia, especially following CD19-directed CAR-T therapy, can cause hypogammaglobulinaemia and increase susceptibility to bacterial, viral, and fungal infections (Zhang et al., 2022; Li et al., 2021; Wei et al., 2020; Hill and Seo, 2020). Management includes immunoglobulin replacement, antimicrobial prophylaxis, vaccination, and monitoring for viral reactivation, particularly hepatitis B virus (Los-Arcos et al., 2021; Jain et al., 2020; Park et al., 2018; Mak et al., 2023; Wei et al., 2019). Infection risk is also influenced by disease burden, corticosteroid use, CRS severity, and delayed immune recovery (Murthy et al., 2019).

Severe CRS may also lead to coagulopathy, including thrombosis, bleeding, and disseminated intravascular coagulation (Johnsrud et al., 2021; Wang et al., 2020; Shao et al., 2021). In addition, prolonged cytopenias may persist because of lymphodepleting chemotherapy, inflammatory cytokines, and impaired bone marrow recovery (Neelapu et al., 2017; Strati et al., 2023; Xing et al., 2021). Current management remains largely supportive.

Antigen mutation, downregulation, and antigen loss are major mechanisms of treatment resistance rather than toxicities. These challenges have driven the development of allogeneic genome-edited CAR-T cells, dual-target CARs, and alternative cellular platforms, although their long-term clinical benefit remains under investigation (Wang et al., 2025; Benjamin et al., 2020; Pan et al., 2023; Shah and Fry, 2019; Xu et al., 2019; Schultz, 2020; Leahy et al., 2021; Hayden et al., 2022; Ganapathy et al., 2023; Mirzaei et al., 2016; Liu et al., 2021; Dai et al., 2020; Li et al., 2023).

Immune effector cell-associated neurotoxicity syndrome (ICANS) presents with manifestations ranging from encephalopathy to cerebral oedema and is associated with endothelial activation and blood–brain barrier disruption (Gust et al., 2017). Although grading systems and supportive care have improved management (Lee et al., 2019; Neelapu et al., 2018), treatment remains largely supportive because reliable predictive biomarkers are lacking (Santomasso et al., 2018; Rubin et al., 2019).

Graft-versus-host disease (GVHD) remains a key concern in allogeneic CAR-T therapies despite advances in genome editing aimed at reducing alloreactivity (Brudno and Kochenderfer, 2016). Future progress will depend on predictive biomarkers, safer CAR designs, improved immune monitoring, and targeted strategies that minimise toxicity while preserving antitumour efficacy.

## 5 Management of toxicities in CAR-T cell therapy

Effective management of CAR-T-cell toxicities is essential to maximise therapeutic benefit while preserving antitumour efficacy. Current approaches emphasise early recognition, standardised grading systems, multidisciplinary care, and prompt intervention to reduce morbidity and treatment-related mortality (Grupp et al., 2013; Hay et al., 2017).

### 5.1 Management of cytokine release syndrome (CRS)

Early intervention is critical for limiting CRS severity. Tocilizumab remains the standard first-line treatment for moderate to severe CRS, whereas corticosteroids are reserved for refractory or severe cases because excessive immunosuppression may reduce CAR-T-cell persistence (Spiegel et al., 2021; Lee et al., 2014). Emerging strategies, including IL-1 blockade with anakinra and GM-CSF inhibition, together with tumour debulking before CAR-T-cell infusion, may further reduce CRS severity, although additional clinical validation is required (Hay et al., 2017; Teachey et al., 2016).

### 5.2 Management of CRS-Associated coagulopathy

Management of CRS-associated coagulopathy relies on routine monitoring of coagulation parameters and individualised supportive care. Blood product replacement, anticoagulation when appropriate, prompt treatment of concurrent infections, and multidisciplinary management remain the principal therapeutic strategies because evidence-based guidelines are still evolving (Park et al., 2018; Lee et al., 2019; Neelapu et al., 2018; Karschnia et al., 2019; Frey et al., 2020).

### 5.3 Management of T- and B-Cell aplasia and infections

Patients with prolonged B-cell aplasia may benefit from immunoglobulin replacement therapy to reduce recurrent or severe infections (Hu, 2019; Kruetzmann et al., 2003; Gupta and Patel, 2020). Long-term management also includes surveillance for bacterial, viral, and fungal infections, antiviral prophylaxis in high-risk patients, and supportive measures to promote immune reconstitution, which remains a significant unmet need following CAR-T-cell therapy (Lee et al., 2019; Grupp et al., 2013).

### 5.4 Management of cytopenias

Persistent cytopenias are managed primarily with supportive measures, including blood transfusions, growth-factor administration, and careful monitoring for infectious complications (Lee et al., 2014; Author Index, 2011; Panitsas et al., 2004). Because the optimal timing of granulocyte colony-stimulating factor remains uncertain, treatment should be individualised while further studies define preventive strategies and mechanisms of prolonged marrow dysfunction (Smith et al., 2015; Turtle et al., 2016).

### 5.5 Management of graft-versus-host disease (GVHD)

In allogeneic CAR-T-cell therapy, GVHD is treated with corticosteroids, calcineurin inhibitors, and selected conditioning strategies, with additional interventions reserved for refractory disease (Penack et al., 2010; Malard and Mohty, 2023). Newer approaches, including genome-edited universal CAR-T cells, natural killer (NK) cells, γδ T cells, and improved donor-cell engineering, aim to minimise GVHD while maintaining durable antitumour activity (Maude et al., 2018; Cohen et al., 2019).

## 6 Target antigens in CAR-T-cell therapy: Current applications, future directions, strengths, and remaining challenges

CAR-T-cell therapy has demonstrated remarkable efficacy in several relapsed or refractory haematological malignancies. However, its long-term success depends largely on selecting appropriate tumour-associated antigens that are highly expressed on malignant cells while showing minimal expression in normal tissues. Antigen heterogeneity and antigen loss remain major obstacles to durable responses.

### 6.1 Importance of targeted antigens

Target-antigen selection is fundamental to CAR-T-cell specificity and efficacy. Characteristics such as antigen density, stability, and restricted expression in normal tissues determine both therapeutic activity and safety. CD19 remains the benchmark target for B-cell malignancies, whereas BCMA has become the principal target in multiple myeloma, although antigen downregulation and loss continue to contribute to disease relapse (Maude et al., 2018; Cohen et al., 2019).

### 6.2 Future perspectives

Current research is focused on overcoming antigen escape and improving CAR-T-cell persistence through dual-target and logic-gated CARs, affinity-optimised receptors, armoured CAR-T cells, and genome-edited allogeneic products (Brudno et al., 2018). These approaches aim to broaden tumour recognition and improve accessibility but require further clinical validation before widespread implementation.

### 6.3 Strengths of CAR-T-cell therapy

CAR-T-cell therapy provides a highly adaptable, living therapeutic platform capable of antigen-specific tumour recognition and prolonged immune surveillance. Advances in receptor engineering and manufacturing continue to improve treatment efficacy across haematological malignancies (Schuster et al., 2019; Munshi et al., 2021).

### 6.4 Limitations of CAR-T-cell therapy

Beyond treatment-related toxicities discussed previously, several biological and practical limitations continue to restrict CAR-T-cell therapy. Antigen escape, tumour heterogeneity, limited CAR-T-cell persistence, and T-cell exhaustion remain major causes of treatment failure (Maude et al., 2018; Cohen et al., 2019; Brudno et al., 2018). In solid tumours, poor tumour infiltration, an immunosuppressive tumour microenvironment, and the scarcity of truly tumour-specific antigens substantially reduce therapeutic efficacy. In addition, autologous CAR-T-cell manufacturing remains costly, labour-intensive, and time-consuming, which may delay treatment for patients with rapidly progressive disease (Brudno et al., 2018; Schuster et al., 2019; Munshi et al., 2021). These challenges highlight the need for improved antigen discovery, predictive biomarkers, more efficient manufacturing platforms, and next-generation CAR designs capable of maintaining durable antitumour activity.

Table 1 Representative CAR-T-cell therapies illustrating the relationship between antigen selection, cancer type, and clinical development (Schuster et al., 2019; Munshi et al., 2021). Although CD19^−^and BCMA-directed products have achieved the strongest results in haematological malignancies, their limitations—including antigen escape, relapse, toxicity, and uncertain long-term persistence—demonstrate that target selection must be considered together with receptor design, tumour biology, and the immune microenvironment.

**TABLE 1:** Representative CAR-T-cell therapies and their target antigens (Schuster et al., 2019; Munshi et al., 2021).

CAR-T-cell therapy	Target antigen	Cancer type	Critical clinical considerations
bb21217	BCMA	Multiple myeloma	Designed to enhance persistence; investigational, with durability and antigen escape requiring further evaluation
CTL019	CD19	B-cell malignancies	Developmental platform underlying an approved CD19-directed therapy; relapse may occur through CD19 loss
CART-PSMA	PSMA	Prostate cancer	Early clinical investigation; limited by tumour heterogeneity, trafficking barriers, and immunosuppression
CAR-EGFRvIII	EGFRvIII	Glioblastoma	Tumour-selective mutation, but heterogeneous expression and antigen loss restrict efficacy
Mesothelin-CAR-T	Mesothelin	Mesothelioma and ovarian cancer	Investigational; stromal barriers, limited persistence, and normal-tissue expression remain concerns
MUC1-CAR-T	MUC1	Multiple epithelial tumours	Glycoform specificity is important because MUC1 is also expressed in normal epithelial tissues
ALLO-501	CD19	Non-Hodgkin lymphoma	Allogeneic platform offering rapid availability, but persistence, host rejection, and lymphodepletion remain challenges
JCARH125	BCMA	Multiple myeloma	Encouraging early activity; long-term efficacy and resistance remain uncertain
CT053	BCMA	Multiple myeloma	Investigational product with promising early responses; larger comparative studies are required

### 6.5 bb21217 CAR-T Cell therapy

bb21217 is a BCMA-directed CAR-T cell therapy developed for relapsed or refractory multiple myeloma. Early phase clinical studies demonstrated encouraging response rates, supporting BCMA as a highly effective therapeutic target. The incorporation of a memory-like T-cell phenotype may enhance persistence and contribute to more durable responses. However, current evidence remains limited by relatively short follow-up, and the durability of remission, long-term persistence, and safety profile require further validation in larger studies. Cytokine release syndrome and neurotoxicity remain important clinical challenges that may limit broader implementation. Although bb21217 represents a promising therapeutic advance, additional clinical evidence is needed to establish its long-term efficacy and define its role in routine practice (Raje et al., 2019a).

### 6.6 CART-PSMA therapy

PSMA-targeted CAR-T (CART-PSMA) therapy has emerged as a promising strategy for metastatic castration-resistant prostate cancer (mCRPC), exploiting the high expression of prostate-specific membrane antigen on tumour cells (McKean et al., 2022). Early-phase clinical trials have demonstrated acceptable safety and preliminary antitumour activity, including partial responses in some patients. However, its clinical efficacy remains limited by several challenges that have hindered successful translation to solid tumours. These include poor T-cell trafficking, antigen heterogeneity, an immunosuppressive tumour microenvironment dominated by TGF-β and inhibitory checkpoint pathways, limited CAR-T persistence, and the risk of tumour escape through reduced or heterogeneous PSMA expression (McKean et al., 2022; Narayan et al., 2022). Furthermore, treatment-related toxicities, particularly cytokine release syndrome and neurotoxicity, continue to require careful management (Zahid et al., 2020). Current research is therefore focused on overcoming these barriers through armored CAR-T cells resistant to TGF-β signalling, combination strategies with PD-1/PD-L1 inhibitors, androgen deprivation therapy or radiotherapy, and improved CAR designs that enhance persistence and infiltration into tumour tissue (Narayan et al., 2022; Serganova et al., 2016). Although these approaches are biologically promising, larger clinical studies are still required to determine whether they can achieve durable clinical benefit in patients with advanced prostate cancer (Lauri et al., 2022).

### 6.7 CAR-EGFRvIII therapy

CAR-EGFRvIII therapy was developed to target the tumour-specific EGFRvIII mutation in glioblastoma, offering greater tumour specificity and reducing the risk of off-tumour toxicity (Sampson et al., 2014). Although preclinical studies demonstrated potent antitumour activity, clinical translation has been disappointing. In a landmark clinical trial, EGFRvIII-directed CAR-T cells successfully trafficked to tumour sites but failed to produce durable clinical responses because treatment induced rapid antigen loss and adaptive resistance, allowing EGFRvIII-negative tumour clones to expand (OʹRourke et al., 2017). Additional barriers, including marked intratumoural heterogeneity, the highly immunosuppressive brain tumour microenvironment, limited CAR-T persistence, and T-cell exhaustion, have further restricted therapeutic efficacy. Consequently, EGFRvIII monotherapy is now considered insufficient for most patients with glioblastoma. Current research focuses on multi-antigen CAR constructs, combination therapy with immune checkpoint inhibitors or radiotherapy, and genetically engineered or armored CAR-T cells designed to resist immunosuppression and enhance persistence. These approaches aim to overcome the biological limitations identified in early clinical studies, but their therapeutic value remains to be confirmed in larger clinical trials (Sampson et al., 2014; OʹRourke et al., 2017).

### 6.8 MUC1-CAR-T Cell therapy

MUC1-directed CAR-T therapy is attractive for epithelial malignancies because tumour-associated MUC1 glycoforms are widely expressed in breast, pancreatic, ovarian, and lung cancers. Preclinical studies using CARs directed against the Tn-glycoform or tumour-associated MUC1 have demonstrated tumour regression, including activity in triple-negative breast cancer models (Posey et al., 2016; Zhou et al., 2019). However, clinical translation remains unproven. The principal limitation is the difficulty of discriminating malignant MUC1 glycoforms from MUC1 expressed on normal epithelial tissues, creating a risk of on-target/off-tumour toxicity. Therapeutic efficacy may also be restricted by heterogeneous antigen density, extensive MUC1 shedding, poor CAR-T trafficking, T-cell exhaustion, and the immunosuppressive microenvironment characteristic of solid tumours. Furthermore, the available evidence is predominantly preclinical, and tumour reduction in animal models does not establish durable benefit in patients. Future research should prioritise highly glycoform-specific binders, affinity-tuned or logic-gated CARs, improved models for predicting epithelial toxicity, and combination strategies that enhance trafficking and resistance to checkpoint- and cytokine-mediated suppression. Early-phase clinical trials will be essential to determine whether MUC1-CAR-T cells can achieve meaningful antitumour activity without unacceptable damage to normal tissues (Posey et al., 2016; Zhou et al., 2019).

### 6.9 Mesothelin-CAR-T Cell therapy

Mesothelin-targeted CAR-T therapy has emerged as a promising strategy for mesothelioma, pancreatic, and ovarian cancers because mesothelin is highly expressed in these malignancies while showing limited expression in normal tissues (Beatty et al., 2018; Adusumilli et al., 2014). Although early-phase clinical studies have demonstrated acceptable safety and evidence of antitumour activity, clinical responses have generally been modest and inconsistent, particularly in advanced solid tumours (Beatty et al., 2018). The principal barriers include poor CAR-T cell trafficking, the dense desmoplastic stroma characteristic of pancreatic cancer, an immunosuppressive tumour microenvironment, limited *in vivo* persistence, and tumour antigen heterogeneity, all of which restrict durable clinical efficacy. Moreover, mesothelin expression in some normal tissues raises concerns regarding on-target/off-tumour toxicity. Current research is therefore focused on multi-target and armored CAR-T constructs, regional delivery, gene-editing strategies, and combination therapies designed to enhance tumour infiltration and overcome immune suppression. Future clinical studies will need to demonstrate durable responses and improved survival before mesothelin-directed CAR-T therapy can be established as an effective treatment for solid tumours (Beatty et al., 2018; Adusumilli et al., 2014).

### 6.10 ALLO-501 therapy

ALLO-501 is an allogeneic, CD19-directed CAR-T product developed to provide an immediately available alternative to patient-specific manufacturing for relapsed or refractory B-cell malignancies. Early ALPHA study results demonstrated clinically meaningful responses in non-Hodgkin lymphoma, supporting the feasibility of an off-the-shelf approach. However, comparisons with autologous CAR-T therapy remain preliminary because the available evidence is derived largely from early-phase, non-randomised studies with limited follow-up. Its broader success may be constrained by host immune rejection, reduced cellular persistence, the need for intensified lymphodepletion, infection risk, prolonged cytopenias, and the potential for graft-versus-host disease despite gene editing. In addition, CD19 loss, impaired CAR-T fitness and other resistance mechanisms may still lead to relapse. Although allogeneic production could reduce manufacturing delays and improve scalability, it does not automatically eliminate cost, logistical or quality-control challenges. Future research should prioritise randomised comparisons with autologous products, optimisation of lymphodepletion, strategies to prevent host rejection and antigen escape, and long-term evaluation of persistence, safety, survival benefit and cost-effectiveness before ALLO-501 can be considered a broadly applicable standard therapy (Roddie et al., 2019; Neelapu et al., 2021; Shah and Fry, 2019).

### 6.11 ALLO-501A therapy

ALLO-501A is a next-generation allogeneic anti-CD19 CAR-T therapy engineered with TCRα and CD52 gene disruption to reduce graft-versus-host disease and permit selective lymphodepletion with ALLO-647 (Locke et al., 2021). Preliminary results from the Phase I ALPHA2 study demonstrated manageable toxicity and early antitumour activity in relapsed or refractory large B-cell lymphoma, with no reported GvHD or immune effector cell-associated neurotoxicity syndrome (Locke et al., 2021). However, clinical efficacy remains uncertain because the study enrolled a small number of patients and follow-up was limited, precluding robust assessment of response durability and long-term safety. As with other allogeneic CAR-T products, challenges include host immune rejection, limited *in vivo* persistence, prolonged cytopenias, infection risk, and the potential for relapse through antigen escape or inadequate CAR-T expansion. Future research should focus on confirming long-term efficacy in larger controlled trials, optimising gene-editing strategies to improve persistence, reducing host-versus-graft immune responses, and determining whether ALLO-501A offers meaningful advantages over established autologous CAR-T therapies (Locke et al., 2021).

### 6.12 JCARH125 therapy

JCARH125 is a BCMA-directed CAR-T therapy developed for relapsed or refractory multiple myeloma, where early findings from the Phase I/II EVOLVE study demonstrated encouraging response rates in heavily pretreated patients. However, these results remain preliminary, and long-term efficacy has yet to be established. As with other BCMA-targeted therapies, durable clinical benefit may be limited by antigen loss or downregulation, CAR-T cell exhaustion, restricted persistence, and disease relapse following initial response. In addition, cytokine release syndrome and neurotoxicity continue to present important safety challenges despite generally manageable toxicity profiles (Raje et al., 2019b; Mailankody et al., 2018). Although BCMA is an attractive therapeutic target with limited off-tumour expression, its optimal clinical application remains uncertain. Future research should focus on improving CAR-T persistence, preventing antigen escape through multi-target or combination approaches, refining toxicity management, and validating long-term survival benefits in larger comparative clinical trials before JCARH125 can be considered a standard treatment for multiple myeloma (Raje et al., 2019b; Mailankody et al., 2018; Abramson, 2020).

### 6.13 CT053 therapy

CT053 is a fully human BCMA-directed CAR-T therapy developed for relapsed or refractory multiple myeloma, with Phase I studies reporting high initial response rates and encouraging progression-free survival in heavily pretreated patients (Hao et al., 2020). Despite these promising findings, the evidence remains limited by the small sample size and early-phase study design, precluding definitive conclusions regarding long-term efficacy. Disease progression in a substantial proportion of treated patients highlights the persistent challenges of antigen escape, limited CAR-T persistence, T-cell exhaustion, and resistance mechanisms that ultimately compromise durable disease control. Although cytokine release syndrome was generally low grade and manageable, haematological toxicities remain common and require careful monitoring (Hao et al., 2020). Future research should prioritise larger randomised trials, extended follow-up to confirm survival benefit, strategies to enhance CAR-T persistence and prevent BCMA escape, and combination or multi-target approaches capable of overcoming relapse and improving long-term clinical outcomes.

## 7 Other cells that belong to the arsenal of CAR-T cells

### 7.1 CD22 CAR-T Cell therapy

CD22-directed CAR-T therapy has emerged as an important salvage strategy for patients with B-cell malignancies who relapse after CD19-targeted CAR-T treatment, particularly through CD19 antigen loss (Frank et al., 2024). Recent clinical studies have demonstrated meaningful responses in relapsed or refractory acute lymphoblastic leukemia and large B-cell lymphoma, confirming CD22 as a clinically relevant target (Frank et al., 2024; Srinagesh et al., 2024). However, durable remission remains challenging because tumour cells may downregulate or lose CD22 expression, resulting in renewed antigen escape. Additional limitations include CAR-T cell exhaustion, limited persistence, infectious complications associated with profound B-cell depletion, and the complexity of treating heavily pretreated patients (Wu et al., 2024a). Consequently, CD22-directed therapy should not be considered a definitive solution to antigen escape but rather part of a broader strategy to overcome resistance. Future research should prioritise dual-target CD19/CD22 constructs, optimisation of CAR design to improve persistence, strategies to reduce infectious complications, and larger comparative clinical trials to determine whether CD22-targeted approaches provide superior long-term outcomes over sequential single-antigen therapies (Frank et al., 2024; Srinagesh et al., 2024; Wu et al., 2024a).

### 7.2 CD20 CAR-T Cell therapy

CD20-directed CAR-T therapy has emerged as a promising salvage strategy for patients with relapsed or refractory B-cell malignancies, particularly following failure of CD19-targeted CAR-T therapy. Early clinical studies have demonstrated encouraging response rates, confirming CD20 as a viable alternative target. However, durable clinical benefit remains uncertain because tumour cells may downregulate CD20 or develop additional resistance mechanisms, leading to disease relapse. Other limitations include limited CAR-T persistence, T-cell exhaustion, and the cumulative effects of prior therapies on immune function. Moreover, evidence is currently derived from small, early-phase studies, limiting conclusions regarding long-term efficacy and survival benefit (Xue et al., 2024). Future research should focus on dual-target CAR-T strategies targeting both CD19 and CD20, optimisation of CAR constructs to improve persistence, rational combinations with immune checkpoint inhibitors, and larger comparative clinical trials to determine whether CD20-directed CAR-T therapy provides sustained advantages over existing therapeutic approaches (Wu et al., 2024b; Xue et al., 2024).

### 7.3 HER2 CAR-T Cell therapy

HER2-directed CAR-T therapy has emerged as a promising approach for HER2-positive solid tumours, including breast, gastric, ovarian, and lung cancers, particularly in patients who have progressed despite HER2-targeted monoclonal antibodies (Budi et al., 2022). Despite encouraging preclinical data and early clinical evidence of antitumour activity, clinical translation has been constrained by the narrow therapeutic window created by HER2 expression in normal tissues, resulting in a significant risk of on-target/off-tumour toxicity. Additional barriers include heterogeneous HER2 expression, poor CAR-T trafficking and persistence, T-cell exhaustion, and the immunosuppressive tumour microenvironment, all of which limit durable clinical responses (Budi et al., 2022). Recent strategies, including affinity-tuned CARs, targeting of membrane-proximal HER2 epitopes, regional delivery, and combination with immune checkpoint inhibitors, aim to improve tumour selectivity while reducing toxicity (Shabaneh et al., 2024; Zhou G. et al., 2024). However, these approaches remain largely investigational, and larger clinical trials are needed to establish long-term safety, durable efficacy, and the optimal role of HER2-directed CAR-T therapy in the treatment of solid tumours (Budi et al., 2022; Shabaneh et al., 2024; Zhou G. et al., 2024). Figure 2 (Zhou G. et al., 2024) shows the molecular docking simulation of anti-HER2 mAbs with homology model of human HER2.

**FIGURE 2:**
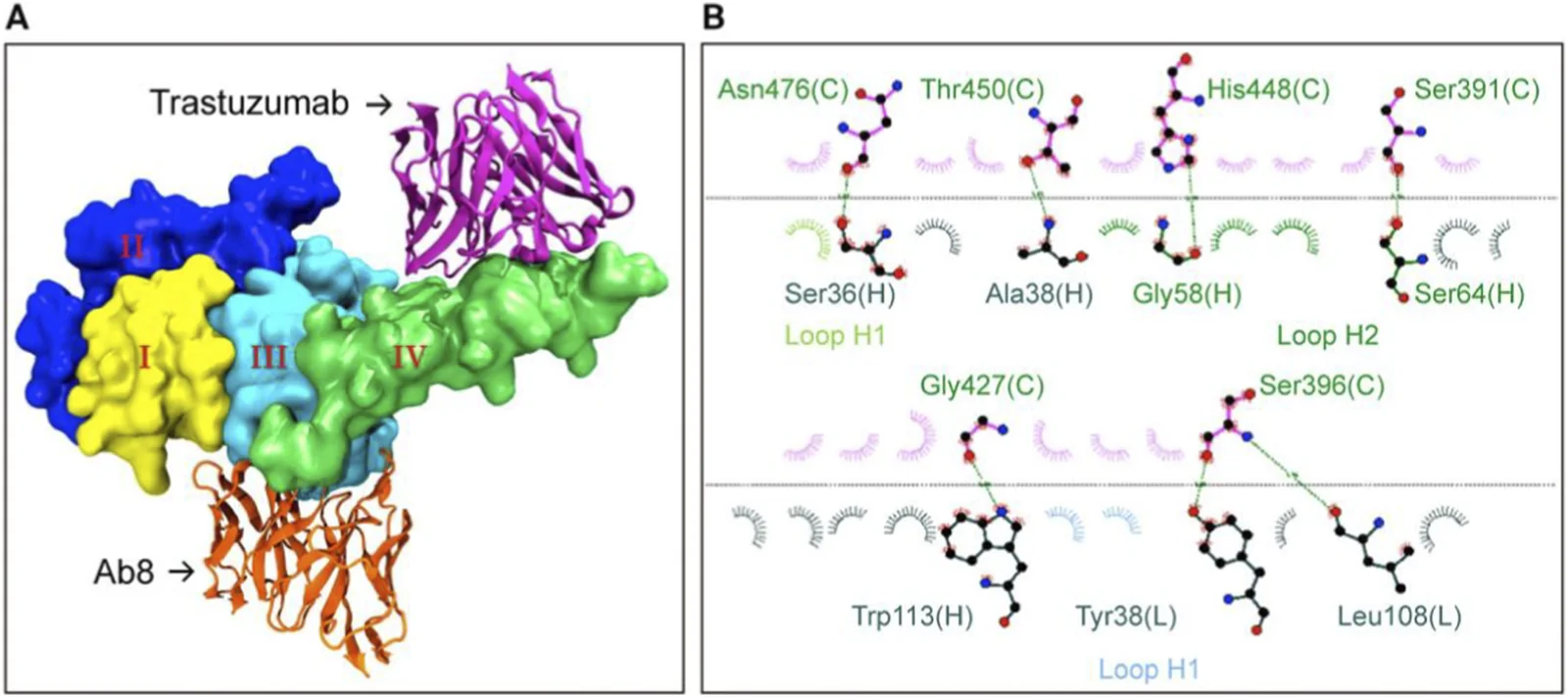
“Molecular docking simulation of anti-HER2 mAbs with homology model of human HER2. **(A)** The docking model of the antibody and antigen. The four domains of Her2 are presented in different colors. The variable regions of heavy and light chains of trastuzumab and Ab8 are represented in violet and orange, respectively. The different domains of HER2 are labelled. **(B)** The predominant residues in the interaction between Ab8 and HER2.” Taken and modified from (Zhou G. et al., 2024).

### 7.4 GD2 CAR-T Cell therapy

GD2-directed CAR-T therapy has shown considerable promise for GD2-positive malignancies, particularly high-risk neuroblastoma, where conventional therapies frequently fail (Locatelli and Quintarelli, 2024). Early-phase clinical studies have demonstrated biological activity and occasional tumour regression in neuroblastoma and other solid tumours. However, durable clinical responses remain limited by several challenges, including heterogeneous GD2 expression, poor CAR-T cell persistence, inadequate trafficking into solid tumours, and the highly immunosuppressive tumour microenvironment (Gargett et al., 2024). In addition, GD2 expression on peripheral nerves predisposes patients to on-target/off-tumour toxicity, including neuropathic pain and neurotoxicity, which restrict the therapeutic window. Recent strategies, including affinity optimisation, improved CAR engineering, and induced pluripotent stem cell-derived GD2 CAR-T cells, aim to enhance persistence, reduce toxicity, and broaden therapeutic applicability (Kinoshita et al., 2024). Nevertheless, these approaches remain investigational, and future studies should prioritise improving long-term efficacy, overcoming resistance mechanisms, and validating safety and survival benefits in larger clinical trials before GD2-directed CAR-T therapy can become a standard treatment for solid tumours (Locatelli and Quintarelli, 2024; Gargett et al., 2024; Kinoshita et al., 2024).

### 7.5 GPC3 CAR-T Cell therapy

GPC3-directed CAR-T therapy has emerged as a promising strategy for hepatocellular carcinoma (HCC) because glypican-3 is highly expressed in HCC while showing limited expression in normal adult tissues (Zhou L. et al., 2024; Zheng et al., 2022). Although preclinical studies and early-phase clinical trials have demonstrated encouraging antitumour activity, clinical translation remains limited. Major barriers include the immunosuppressive liver tumour microenvironment, poor CAR-T cell trafficking and persistence, tumour heterogeneity, and the potential for on-target/off-tumour toxicity. Current research is focused on bispecific CAR-T cells targeting GPC3 and fibroblast activation protein (FAP), BiTE-secreting CAR-T cells, and combination strategies designed to improve tumour infiltration and overcome immune suppression (Zhou L. et al., 2024; Cao et al., 2022). Future studies should prioritise larger clinical trials, predictive biomarkers of response, and long-term safety and survival before GPC3-directed CAR-T therapy can become a standard treatment for HCC and other GPC3-expressing solid tumours (Zhou L. et al., 2024; Zheng et al., 2022; Cao et al., 2022; Ortiz et al., 2019).

### 7.6 ROR1 (receptor tyrosine kinase-like orphan receptor 1) CAR-T Cell therapy

ROR1-directed CAR-T therapy has emerged as a promising strategy for haematological and solid malignancies because ROR1 is an oncofetal antigen with restricted expression in normal adult tissues but aberrant expression in chronic lymphocytic leukaemia, mantle cell lymphoma, breast cancer, colorectal cancer, and other malignancies (Yin et al., 2023; Meng et al., 2023). Although preclinical studies have demonstrated potent antitumour activity, clinical translation remains at an early stage, and durable clinical efficacy has yet to be established. Major barriers include the immunosuppressive tumour microenvironment, limited CAR-T cell persistence, tumour heterogeneity, and the potential for on-target/off-tumour toxicity resulting from low-level ROR1 expression in normal tissues. These factors may restrict long-term therapeutic benefit despite encouraging preclinical results. Current research is focused on armored ROR1 CAR-T cells resistant to TGF-β-mediated immunosuppression, optimisation of CAR design, and combination strategies to improve persistence and tumour infiltration (Tran et al., 2024). Future studies should prioritise validating these approaches in larger clinical trials, identifying predictive biomarkers, and establishing long-term safety and survival benefits before ROR1-directed CAR-T therapy can be broadly implemented across multiple cancer types (Yin et al., 2023; Meng et al., 2023; Tran et al., 2024).

### 7.7 NY-ESO-1 CAR-T Cell therapy

NY-ESO-1-directed CAR-T therapy targets a cancer-testis antigen expressed in synovial sarcoma, melanoma, multiple myeloma, hepatocellular carcinoma, and other malignancies, making it an attractive target because of its limited expression in normal tissues (Liu X. et al., 2022; Rochigneux et al., 2021). Although preclinical studies and early-phase clinical trials have demonstrated encouraging antitumour activity, particularly in synovial sarcoma, clinical translation remains limited. Durable responses are constrained by tumour heterogeneity, antigen loss, restricted CAR-T cell persistence, and the immunosuppressive tumour microenvironment, which promotes T-cell dysfunction and limits tumour infiltration. Moreover, because NY-ESO-1 recognition is generally HLA-restricted, patient eligibility is limited compared with conventional CAR-T therapies, reducing its broader applicability (Liu X. et al., 2022). Current research is focused on improving CAR or TCR-like receptor design, enhancing persistence through combination therapies, and overcoming immune suppression within the tumour microenvironment. Future studies should validate these strategies in larger clinical trials and determine whether they provide durable survival benefits across different NY-ESO-1-positive malignancies before widespread clinical implementation (Liu X. et al., 2022; Rochigneux et al., 2021).

### 7.8 NKG2D CAR-T Cell therapy

NKG2D-directed CAR-T therapy represents an alternative to conventional single-antigen CAR-T approaches by targeting multiple stress-induced ligands, including MICA, MICB, and ULBPs, which are expressed across a broad range of haematological and solid malignancies (Mai et al., 2024; Wang D. et al., 2024; Fuertes et al., 2021). This multi-ligand recognition may reduce, but not eliminate, antigen escape associated with tumour heterogeneity. Although preclinical studies have demonstrated encouraging antitumour activity, clinical translation remains limited. A major challenge is the inducible expression of NKG2D ligands on normal tissues during inflammation, increasing the risk of on-target/off-tumour toxicity. In addition, ligand shedding, the immunosuppressive tumour microenvironment, limited CAR-T persistence, and the inability to eradicate tumour-initiating cells have restricted durable therapeutic responses (Fuertes et al., 2021; Ibáñez-Navarro et al., 2023). Current research is focused on cytokine-enhanced constructs incorporating IL-15 or IL-21, combination immunotherapies, and optimisation of CAR engineering to improve persistence and resistance to immune suppression (Mai et al., 2024; Zhang et al., 2024). Future clinical studies should determine whether these modifications can achieve sustained tumour control while maintaining an acceptable safety profile across diverse malignancies (Mai et al., 2024; Wang D. et al., 2024; Fuertes et al., 2021; Zhang et al., 2024; Ibáñez-Navarro et al., 2023).

## 8 Methods for studying the binding of CAR-T cells to antigens and their importance

The interaction between CAR-T cells and their target antigens is a key determinant of therapeutic efficacy, persistence, and toxicity. Consequently, accurate characterisation of CAR-antigen binding is essential for optimising CAR design, predicting clinical responses, and identifying mechanisms of treatment failure. Although several analytical techniques are available, each provides only partial information, highlighting the need for complementary approaches and more physiologically relevant models (Zhang et al., 2022; Schuster et al., 2019; Sang et al., 2020).

### 8.1 Current methods for studying CAR-T Cell binding

Several experimental platforms are used to evaluate CAR-T cell–antigen interactions. Surface plasmon resonance (SPR) provides quantitative real-time measurements of binding affinity and association/dissociation kinetics but is performed under cell-free conditions and therefore does not fully reproduce the complexity of immune synapse formation *in vivo* (Mallis et al., 2019). Flow cytometry remains the most widely used method for assessing antigen recognition and binding specificity at the single-cell level; however, it measures binding efficiency rather than functional cytotoxicity or long-term CAR-T persistence (Shepherd et al., 2024). ELISA offers a simple, high-throughput approach for comparing CAR constructs but lacks the ability to evaluate dynamic cellular interactions and therefore has limited physiological relevance (Potthoff et al., 2020). In contrast, confocal and fluorescence microscopy provide detailed visualisation of immune synapse formation and CAR-antigen spatial organisation, although these techniques are technically demanding, low throughput, and primarily descriptive (Wang Z. et al., 2024). Collectively, no single method comprehensively predicts therapeutic performance, and integrating complementary assays is increasingly recommended.

### 8.2 Importance of characterising CAR-T Cell binding

Detailed analysis of CAR-antigen interactions is essential for balancing affinity, specificity, and safety during CAR optimisation. Excessively high-affinity CARs may increase on-target/off-tumour toxicity, whereas low-affinity receptors may compromise antitumour efficacy (Ghorai and Pearson, 2024). Furthermore, binding characteristics have been associated with treatment response, persistence, and durability of remission, suggesting potential value as predictive biomarkers, although clinical validation remains limited (Cappell and Kochenderfer, 2023). Characterising binding also improves understanding of resistance mechanisms, including antigen loss, antigen modulation, and tumour heterogeneity, thereby supporting the development of dual-target, logic-gated, and combination CAR-T strategies (Srivastava and Riddell, 2018).

### 8.3 Future perspectives

Current analytical platforms remain largely *in vitro* and do not fully capture the complexity of the tumour microenvironment. Emerging technologies, including single-cell multi-omics, may improve understanding of functional heterogeneity within CAR-T cell populations (Huang et al., 2023). Advances in in vivo molecular imaging could enable real-time monitoring of CAR-T trafficking, persistence, and tumour engagement, facilitating earlier assessment of therapeutic response (Si et al., 2022). In addition, artificial intelligence and machine learning are increasingly being applied to integrate multidimensional datasets, identify predictive biomarkers, optimise CAR design, and personalise patient selection. Nevertheless, these computational approaches require rigorous prospective validation before routine clinical implementation (Hort et al., 2022).

## 9 Advancements in NK CAR cells for cancer immunotherapy

### 9.1 Development of NK CAR cells

The limitations of conventional CAR-T cell therapy, particularly severe treatment-related toxicities, patient-specific manufacturing, and limited efficacy against solid tumours, have stimulated the development of chimeric antigen receptor-engineered natural killer (CAR-NK) cells as an alternative adoptive immunotherapy platform. Natural killer (NK) cells are innate lymphocytes that recognise and eliminate malignant cells independently of major histocompatibility complex (MHC) restriction, allowing them to detect tumour cells that evade conventional T-cell immunity through downregulation of antigen presentation pathways (Portale and Di Mitri, 2023; Yu, 2023). In addition to their direct cytotoxic activity, NK cells interact extensively with dendritic cells, macrophages, T lymphocytes, and other components of the tumour microenvironment, thereby influencing both innate and adaptive antitumour immune responses (Zhou et al., 2023). Engineering NK cells with CAR constructs seeks to combine these intrinsic biological properties with enhanced antigen specificity while reducing the risks of graft-versus-host disease (GVHD), cytokine release syndrome (CRS), and immune effector cell-associated neurotoxicity syndrome (ICANS) that are more frequently associated with CAR-T therapy (Wang et al., 2024c). Although CAR-NK cells offer the prospect of readily available “off-the-shelf” cellular products with an improved safety profile, their clinical development continues to be challenged by limited *in vivo* persistence, inefficient expansion, and the immunosuppressive tumour microenvironment, particularly in solid malignancies (Portale and Di Mitri, 2023; Yu, 2023; Zhou et al., 2023; Wang et al., 2024c).

Early NK CAR constructs were adapted from CAR-T platforms and successfully demonstrated antitumour activity against CD19-positive haematological malignancies in preclinical studies (Uscanga-Palomeque et al., 2023; Li T. et al., 2024; Bashiri Dezfouli et al., 2021; Daher et al., 2021; Li H. et al., 2022). Subsequent generations have incorporated NK-specific signalling domains, including NKG2D-based activation motifs, together with cytokines such as IL-15 to improve proliferation, cytotoxicity, and *in vivo* persistence (Kong et al., 2024; Ma et al., 2022). Although these modifications have enhanced preclinical efficacy, durable clinical responses remain limited, and the optimal CAR design for NK cells has not yet been established. Figure 3 depicts soluble factors in the bi-directional crosstalk between NK cells and immune cells in the TME.

**FIGURE 3:**
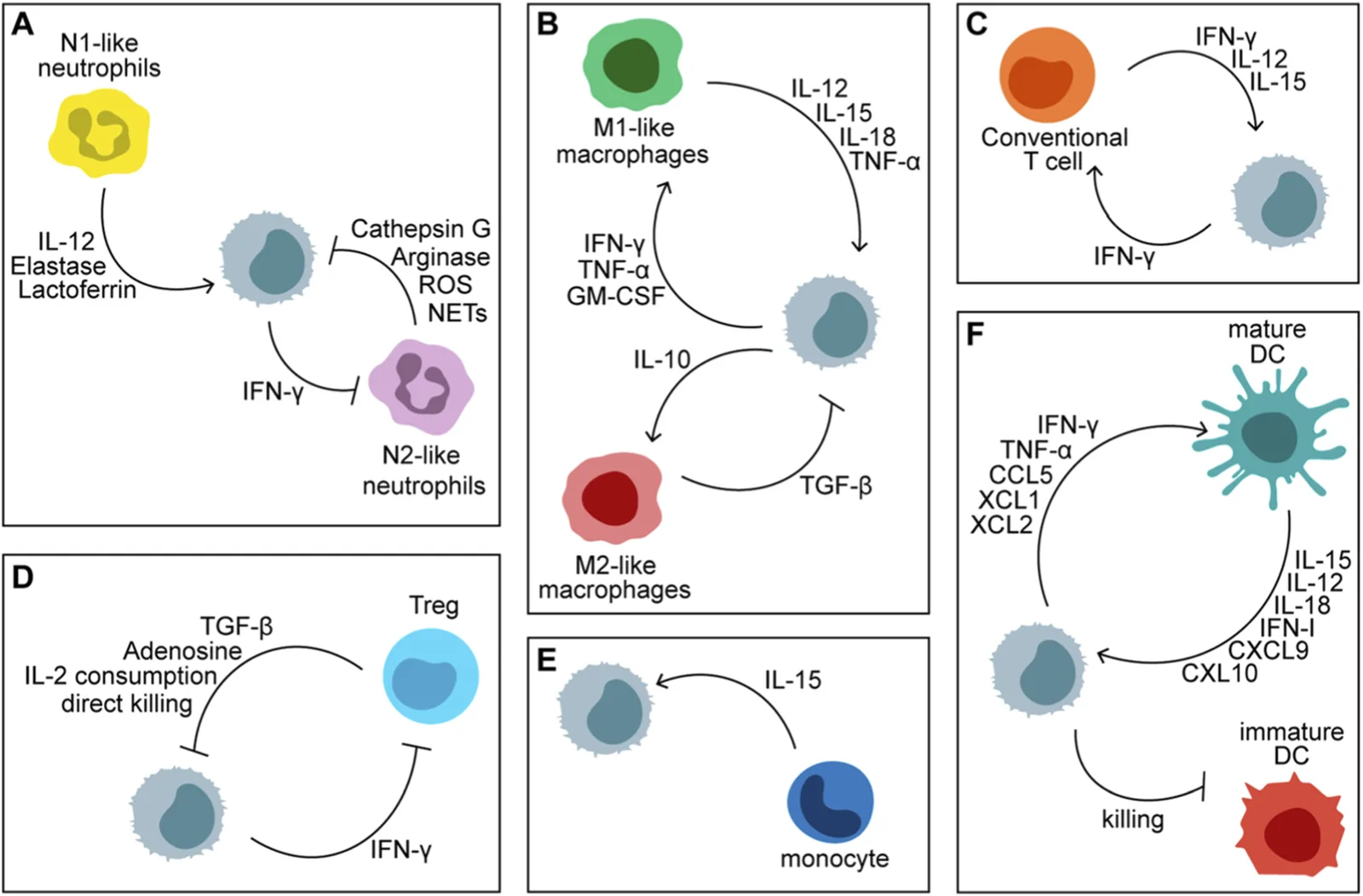
Soluble Factors in the Bi-Directional Crosstalk between NK Cells and Immune Cells in the Tumor Microenvironment (TME). Taken and modified from (Portale and Di Mitri, 2023). NK cellsʹ anti-tumor activities are influenced by a range of cytokines and soluble factors produced by immune cells within the TME, which can either enhance or suppress their functions. **(A)** Neutrophils: Neutrophils can inhibit NK cell function by downregulating NKp46 expression through the release of Cathepsin G and reactive oxygen species (ROS). They also hinder NK cell activity by depleting arginine and releasing neutrophil extracellular traps (NETs), which block IFN-γ production and cytotoxicity. On the other hand, neutrophil-derived elastase, lactoferrin, and interleukin (IL)-12 boost NK cell functions. Additionally, IFN-γ produced by NK cells can suppress the angiogenic potential of tumor-associated neutrophils. **(B)** Macrophages: M1-polarized macrophages in the tumor microenvironment activate NK cells by secreting cytokines such as IL-12, IL-15, IL-18, and TNF-α. In contrast, M2-polarized macrophages inhibit NK cell activation through the release of TGF-β. NK cells, in turn, can modulate macrophage activity by releasing IFN-γ, TNF-α, GM-CSF, and IL-10. **(C)** T Cells: NK cell-derived IFN-γ promotes the polarization of CD4^+^ T cells towards a Th1 response and enhances the cytotoxic functions of CD8^+^ T cells. In response, T cells produce IL-2 and IL-15, which are essential for NK cell survival and activation. **(D)** Regulatory T Cells (Treg): NK cell-mediated tumor killing and IFN-γ production are negatively impacted by immunosuppressive molecules like TGF-β and adenosine, which are released by Tregs. Tregs also suppress NK cell functions by competing for IL-2 and can directly kill NK cells through granzyme B **(E)** Monocytes: Patrolling monocytes contribute to NK cell-mediated anti-metastatic activity by releasing IL-15. **(F)** Dendritic Cells (DCs): NK cell interaction with dendritic cells leads to mutual activation. Mature DCs secrete cytokines such as IL-12, IL-15, IL-18, CXCL9, and CXCL10, which promote NK cell activation and recruitment. Furthermore, NK cells help optimize DC-driven immune responses by eliminating immature DCs. In return, NK cells facilitate the activation and recruitment of DCs within the tumor bed by releasing IFN-γ, TNF-α, CCL5, XCL1, and XCL2. This content was partly created using BioRender.com.

### 9.2 Current challenges

Several biological and technical barriers continue to limit the clinical translation of NK CAR therapy. Efficient genetic modification of NK cells remains more difficult than for T cells, resulting in variable CAR expression and manufacturing challenges despite improvements in viral, non-viral, and transposon-based engineering methods (Schmidt et al., 2022; Matosevic, 2018). In addition, NK cells generally exhibit shorter *in vivo* persistence than CAR-T cells, often necessitating cytokine support or repeated infusions to maintain therapeutic activity (Islam et al., 2021; Tarannum et al., 2022).

The tumour microenvironment remains another major obstacle. Immunosuppressive mediators, checkpoint molecules, regulatory T cells, and myeloid-derived suppressor cells reduce NK-cell activation, impair tumour infiltration, and promote functional exhaustion, particularly in solid tumours (Lu et al., 2023). Consequently, current research increasingly focuses on combining NK CAR therapy with checkpoint inhibitors and other immune-modulating agents to overcome these barriers (Zhong and Liu, 2024).

### 9.3 Clinical translation

One of the principal advantages of NK CAR therapy is the possibility of developing off-the-shelf allogeneic cell products derived from healthy donors or established NK-cell lines, potentially reducing manufacturing time and cost compared with autologous CAR-T therapy (Lu et al., 2021; Li W. et al., 2024). Early clinical trials targeting CD19 have demonstrated favourable safety profiles and encouraging responses in patients with relapsed or refractory B-cell malignancies, including some previously treated with CAR-T therapy (Keshavarz et al., 2022; Rossi et al., 2022). However, these studies remain small, follow-up is limited, and durable efficacy has not yet been confirmed. Clinical evaluation has also expanded to targets such as GD2 and HER2 for solid tumours, although therapeutic benefit has thus far been modest (Fares et al., 2023; Zhong and Liu, 2024; Valeri et al., 2022).

### 9.4 Future perspectives

Future progress will depend on improving NK-cell persistence, resistance to tumour-induced immunosuppression, and manufacturing efficiency. Multi-specific CAR constructs may reduce antigen escape by simultaneously targeting multiple tumour-associated antigens (Wang et al., 2024c). In parallel, combination strategies incorporating checkpoint blockade, cytokine support, and other immunomodulatory approaches are expected to enhance NK-cell persistence and antitumour activity (Li et al., 2024c). Nevertheless, large randomised clinical trials with long-term follow-up remain necessary to determine whether NK CAR therapy can achieve durable clinical outcomes comparable to or exceeding those of established CAR-T therapies.

## 10 The potential of γδ T Cells in mitigating graft-versus-host disease

The development of universal allogeneic CAR-T therapies has intensified interest in alternative immune-cell populations capable of reducing graft-versus-host disease (GVHD) while preserving potent antitumour activity. Among these, γδ T cells have emerged as one of the most promising candidates because they recognise stressed or transformed cells independently of major histocompatibility complex (MHC) presentation, thereby substantially reducing the risk of alloreactivity associated with conventional αβ T cells (Allemailem et al., 2023; Lv et al., 2023). Recent advances in genome-editing technologies, including CRISPR/Cas9, have further facilitated the development of engineered γδ CAR-T cells with improved specificity and the potential for off-the-shelf applications (Allemailem et al., 2023).

Beyond their reduced propensity to induce GVHD, γδ T cells possess several biological characteristics that make them attractive for CAR-based immunotherapy. They exhibit intrinsic cytotoxicity against a broad spectrum of malignant cells, produce pro-inflammatory cytokines that promote antitumour immunity, and demonstrate enhanced tumour-homing capabilities, particularly within solid tumours where conventional CAR-T cells often show limited efficacy (Tang and Zhang, 2024; Yazdanifar et al., 2020). In addition, γδ T cells may contribute to immune surveillance independently of CAR-mediated antigen recognition, potentially reducing the risk of tumour escape caused by antigen loss.

Despite these advantages, clinical translation remains at an early stage. Efficient *ex vivo* expansion, phenotypic heterogeneity among γδ T-cell subsets, limited *in vivo* persistence, and incomplete understanding of their long-term immunological behaviour continue to represent important barriers to widespread clinical application (Tang and Zhang, 2024). Furthermore, although preclinical studies have demonstrated encouraging antitumour activity with a favourable safety profile, robust clinical evidence confirming durable responses and superiority over conventional CAR-T therapies is still lacking (Yazdanifar et al., 2020). Consequently, current research focuses on optimising manufacturing protocols, enhancing persistence through genetic engineering, and integrating γδ T cells with next-generation CAR designs to improve efficacy while maintaining their low risk of GVHD (Allemailem et al., 2023; Lv et al., 2023; Tang and Zhang, 2024; Yazdanifar et al., 2020).

## 11 Emerging cellular platforms and engineering strategies for next-generation CAR therapies

Universal CAR therapies are being developed to overcome the limitations of autologous CAR-T cells, including lengthy manufacturing, high costs, product variability, and restricted patient accessibility. Alternative immune-cell platforms—including TCR-knockout T cells, natural killer T (NKT) cells, double-negative (DN) T cells, macrophages, neutrophils, mucosal-associated invariant T (MAIT) cells, and stromal-targeting CARs—have demonstrated encouraging preclinical activity. However, despite their biological advantages, most remain in early clinical development, and durable efficacy, manufacturing scalability, and long-term safety have yet to be established (Lonez and Breman, 2024).

### 11.1 TCR knockout T Cells

TCR-knockout conventional T cells are designed to eliminate endogenous T-cell receptor expression, thereby reducing the risk of graft-versus-host disease (GVHD) and facilitating universal allogeneic therapy (Caldwell et al., 2021). Advances in CRISPR/Cas gene editing have improved the feasibility of this approach (Song et al., 2024). Nevertheless, manufacturing remains technically complex, concerns regarding off-target genome editing and chromosomal rearrangements persist, and long-term clinical safety has not yet been fully established. Although these engineered cells retain antitumour activity in preclinical models, larger clinical studies are required to determine whether they provide durable efficacy while reducing production costs and expanding patient access (Courtney et al., 2023; Hillhouse et al., 2013; Wu et al., 2022; Liu XC. et al., 2024; Wei W. et al., 2023; Rezalotfi et al., 2022; Zhang et al., 2023).

### 11.2 Natural killer T (NKT) cells

Natural killer T (NKT) cells possess characteristics of both NK cells and conventional T cells (Courtney et al., 2023), allowing rapid immune activation with a relatively low risk of GVHD, making them attractive candidates for universal CAR therapy (Hadiloo et al., 2023a). Their immunoregulatory properties and capacity to modulate the tumour microenvironment may offer advantages over conventional CAR-T cells, particularly in solid tumours (Shissler et al., 2016; Liu Y. et al., 2022; Fujii and Shimizu, 2017). However, clinical translation remains limited by their low frequency in peripheral blood, difficulties in large-scale expansion, and the lack of robust long-term clinical data. Future studies should focus on scalable manufacturing and validating efficacy in prospective clinical trials.

### 11.3 Invariant natural killer T (iNKT) cells

Invariant natural killer T (iNKT) cells have emerged as a promising platform for universal CAR-based immunotherapy because they combine direct cytotoxic activity with potent immunomodulatory functions while exhibiting minimal risk of graft-versus-host disease (GVHD) in the allogeneic setting (Liu Y. et al., 2022; Fujii and Shimizu, 2017). Their ability to infiltrate tumours, activate dendritic cells, and reshape the tumour microenvironment may provide advantages over conventional CAR-T cells, particularly in solid malignancies (Fujii and Shimizu, 2017). However, despite encouraging preclinical findings, clinical translation remains limited by the low frequency of iNKT cells in peripheral blood, difficulties in large-scale expansion, and the absence of robust clinical evidence demonstrating durable efficacy. Consequently, current research focuses on improving manufacturing, enhancing persistence and tumour homing, and validating the long-term safety and therapeutic benefit of CAR-iNKT cells in prospective clinical trials (Liu Y. et al., 2022; Fujii and Shimizu, 2017).

### 11.4 Double-negative (CD4^−^CD8^−^) T Cells

Double-negative T cells (Hillhouse et al., 2013; Wu et al., 2022; Liu XC. et al., 2024) represent another promising universal CAR platform because they demonstrate intrinsic antitumour activity while exhibiting minimal alloreactivity (Provine and Klenerman, 2020; Fischer et al., 2005; Brandt and Hedrich, 2018; Ye et al., 2011). Preclinical studies suggest that CAR-engineered DN T cells can inhibit tumour growth without significant off-target toxicity (Vasic et al., 2022). Despite these encouraging findings, their biology remains incompletely understood, expansion protocols are inefficient, and clinical experience is extremely limited. Further investigation is needed to determine their persistence, safety, and therapeutic durability in patients.

### 11.5 CAR macrophages

CAR macrophages (CAR-M) have emerged as an attractive strategy for solid tumours because macrophages naturally infiltrate tumour tissue and actively remodel the tumour microenvironment (Hadiloo et al., 2023b; Basak et al., 2023). In addition to directly phagocytosing tumour cells, CAR-Ms function as antigen-presenting cells capable of stimulating broader antitumour immunity (Li N. et al., 2024; Sadri et al., 2024; Pan et al., 2022). Nevertheless, clinical translation remains in its infancy owing to manufacturing challenges, limited persistence, macrophage plasticity, and the potential for excessive inflammatory responses. Current research focuses on optimising CAR design and validating safety and efficacy in early clinical studies (Yu et al., 2024; Dong et al., 2023; Chen K. et al., 2024).

### 11.6 CAR neutrophils

CAR-engineered neutrophils derived from pluripotent stem cells represent a novel myeloid-cell platform with the ability to infiltrate difficult tumour sites, including glioblastoma, and deliver targeted therapeutic payloads (Chang et al., 2023). Improvements in stem-cell engineering have enabled scalable production and enhanced antitumour activity against malignancies such as prostate cancer (Harris et al., 2023). However, their naturally short lifespan, manufacturing complexity, and complete absence of clinical data currently limit therapeutic application. Future research should prioritise improving persistence and confirming efficacy in human trials (Chang et al., 2022).

### 11.7 CD5 knockout CAR-T cells

CD5 knockout has emerged as a promising strategy to improve CAR-T therapy for T-cell malignancies by preventing fratricide and enhancing CAR-T cell persistence (Patel et al., 2024). Optimised biepitopic CD5-targeting CARs have demonstrated improved antitumour activity and reduced cytokine release in preclinical studies (Dai et al., 2021). Nevertheless, these findings remain experimental, and additional studies are required to evaluate long-term genomic safety, manufacturing feasibility, and clinical efficacy.

### 11.8 MAIT cells

Mucosal-associated invariant T (MAIT) cells possess innate-like and adaptive immune functions and contribute to antitumour immunity through direct cytotoxicity and modulation of other immune populations within the tumour microenvironment (Chen et al., 2017; Li YR. et al., 2022; Chengalroyen, 2023; Legoux et al., 2020). Engineered CAR-MAIT cells have demonstrated encouraging preclinical cytotoxicity comparable to conventional CAR-T cells against lymphoma and breast cancer models (Dogan et al., 2022). However, their clinical development remains at an early stage, and important questions regarding persistence, trafficking, functional exhaustion, and long-term safety remain unresolved.

### 11.9 Fibroblast activation protein (FAP)-targeted CAR-T cells

FAP-targeted CAR-T cells differ from conventional CAR therapies by targeting cancer-associated fibroblasts within the tumour stroma rather than tumour cells directly (Ruixin et al., 2024; Liu et al., 2023). This approach may enhance immune-cell infiltration, reduce myeloid-derived suppressor cell recruitment, and improve the efficacy of tumour-directed CAR-T therapies (Liu et al., 2023). Despite these advantages, FAP expression in selected normal tissues raises concerns regarding on-target/off-tumour toxicity, and consistent clinical benefit has not yet been demonstrated. Current research therefore focuses on dual-target CAR constructs, sequential therapeutic strategies, and combination approaches designed to maximise efficacy while limiting toxicity (Ruixin et al., 2024; Liu et al., 2023).

### 11.10 Future perspectives

Collectively, these emerging cellular platforms have the potential to broaden the applicability of adoptive cell therapy beyond conventional autologous CAR-T cells. Nevertheless, most remain experimental, with limited clinical evidence demonstrating durable responses. Common challenges include manufacturing complexity, limited persistence, immune suppression within the tumour microenvironment, uncertain long-term safety, and the absence of large comparative clinical trials. Future research should prioritise scalable manufacturing, precision gene editing, improved resistance to tumour-induced immune suppression, and rigorous prospective studies to identify which universal CAR platforms can deliver meaningful and sustained clinical benefit.

### 11.11 Universal CAR-T Cell systems

Universal CAR-T-cell systems have emerged as a promising strategy to overcome several limitations associated with conventional autologous CAR-T-cell therapy, including lengthy manufacturing times, high production costs, product-to-product variability, and limited patient accessibility. By combining gene-editing technologies with alternative immune-cell platforms, these approaches seek to generate readily available “off-the-shelf” cellular therapies while reducing the risk of graft-versus-host disease (GVHD) and preserving potent antitumour activity. Current universal platforms include T-cell receptor (TCR)-knockout αβ T cells, natural killer (NK) cells, invariant natural killer T (iNKT) cells, double-negative (DN) T cells, macrophages, neutrophils, mucosal-associated invariant T (MAIT) cells, and other engineered immune-cell populations. Collectively, these strategies represent a shift from patient-specific manufacturing towards scalable, standardised cellular products capable of broader clinical implementation, although most remain in preclinical or early clinical evaluation (Lonez and Breman, 2024; Caldwell et al., 2021; Song et al., 2024; Courtney et al., 2023; Hillhouse et al., 2013; Wu et al., 2022; Liu XC. et al., 2024; Wei W. et al., 2023; Rezalotfi et al., 2022; Zhang et al., 2023; Hadiloo et al., 2023a; Shissler et al., 2016; Liu Y. et al., 2022; Fujii and Shimizu, 2017). Despite their considerable potential, universal CAR systems continue to face important biological and translational challenges. Although genome-editing technologies have substantially reduced alloreactivity and improved the feasibility of allogeneic products, concerns regarding off-target genetic modifications, host immune rejection, limited *in vivo* persistence, manufacturing consistency, and long-term safety have not yet been fully resolved (Caldwell et al., 2021; Song et al., 2024; Courtney et al., 2023; Hillhouse et al., 2013; Wu et al., 2022; Liu XC. et al., 2024; Wei W. et al., 2023; Rezalotfi et al., 2022; Zhang et al., 2023). Likewise, several alternative cellular platforms demonstrate intrinsic biological advantages, including reduced GVHD risk, improved tumour homing, and immunomodulatory activity, but difficulties in large-scale expansion, phenotypic heterogeneity, and the lack of robust long-term clinical evidence continue to limit widespread adoption (Hadiloo et al., 2023a; Shissler et al., 2016; Liu Y. et al., 2022; Fujii and Shimizu, 2017; Provine and Klenerman, 2020; Fischer et al., 2005; Brandt and Hedrich, 2018; Ye et al., 2011; Vasic et al., 2022). Consequently, future research should prioritise optimisation of manufacturing protocols, enhancement of cellular persistence and resistance to immune suppression, refinement of genome-editing technologies, and well-designed comparative clinical trials to determine whether universal CAR platforms can ultimately provide durable efficacy, improved safety, and greater accessibility than current autologous CAR-T-cell therapies (Lonez and Breman, 2024; Caldwell et al., 2021; Song et al., 2024; Courtney et al., 2023; Hillhouse et al., 2013; Wu et al., 2022; Liu XC. et al., 2024; Wei W. et al., 2023; Rezalotfi et al., 2022; Zhang et al., 2023; Hadiloo et al., 2023a; Shissler et al., 2016; Liu Y. et al., 2022; Fujii and Shimizu, 2017; Provine and Klenerman, 2020; Fischer et al., 2005; Brandt and Hedrich, 2018; Ye et al., 2011; Vasic et al., 2022).

## 12 Antigen mutation and alternative cellular platforms: Opportunities and limitations of γδ CAR-T cells

Antigen escape is one of the major causes of resistance to CAR-T cell therapy. Under selective immune pressure, tumour cells can lose or downregulate target antigens through genetic mutations, alternative RNA splicing, or lineage switching, rendering them resistant to CAR-mediated recognition (Luo et al., 2024). This phenomenon has been well documented in CD19-directed CAR-T therapy for B-cell malignancies and has also emerged with BCMA-targeted therapies in multiple myeloma. Similar mechanisms are anticipated in solid tumours because of their marked genomic instability and intratumoural heterogeneity, highlighting the need for cellular platforms capable of recognising tumour cells independently of a single antigen.

γδ T cells have emerged as a promising alternative because they recognise stressed or transformed cells largely independently of major histocompatibility complex (MHC) presentation. Their intrinsic ability to detect phosphoantigens and stress-induced ligands complements CAR-mediated antigen recognition, theoretically reducing the likelihood of tumour escape following antigen loss (Ansari et al., 2026). In addition, γδ T cells possess innate-like cytotoxicity, secrete interferon-γ and tumour necrosis factor-α, and have a lower propensity to induce graft-versus-host disease, making them attractive candidates for allogeneic “off-the-shelf” CAR-T products that could improve treatment accessibility.

Despite these advantages, several major limitations remain unresolved. Perhaps the greatest obstacle is their inefficient *ex vivo* expansion. γδ T cells account for only 1%–5% of circulating T lymphocytes, and although agents such as zoledronic acid combined with interleukin-2 can selectively expand Vγ9Vδ2 cells, expansion is highly donor-dependent and often fails to generate clinically sufficient cell numbers (Wang et al., 2026). This variability complicates manufacturing, increases production costs, and limits reproducibility across treatment centres.

Another important controversy concerns the biological heterogeneity of γδ T-cell subsets. Circulating Vγ9Vδ2 and tissue-resident Vδ1 cells exhibit distinct functional properties, migratory patterns, and proliferative capacities, yet the optimal subset for CAR engineering remains uncertain. Consequently, inconsistent cell composition may contribute to the variable efficacy observed in preclinical and early clinical studies. Functional persistence is also a major concern. Compared with conventional αβ CAR-T cells, γδ CAR-T cells generally demonstrate reduced long-term expansion and persistence *in vivo*, potentially limiting durable tumour surveillance and increasing the risk of relapse (Peter et al., 2025). Furthermore, despite their innate immune characteristics, they remain susceptible to the immunosuppressive tumour microenvironment, including transforming growth factor-β, interleukin-10, regulatory T cells, myeloid-derived suppressor cells, and checkpoint pathways such as PD-1, all of which can impair proliferation and cytotoxicity, particularly in solid tumours.

Early-phase clinical trials indicate favourable safety profiles, including lower risks of graft-versus-host disease and severe cytokine release syndrome. However, evidence of clinical efficacy remains limited, with most data derived from small Phase I studies. Thus, although γδ CAR-T cells offer a compelling strategy to overcome antigen escape, their future clinical role will depend on overcoming challenges related to expansion, persistence, subset selection, and scalable manufacturing.

## 13 CAR T-Cell therapy for solid tumors: clinical challenges, trial outcomes, and lessons learned

Although CAR T-cell therapy has transformed the treatment of B-cell malignancies, its success has not been replicated in solid tumours. Unlike haematological cancers, solid tumours are characterised by heterogeneous antigen expression, dense stromal barriers, immunosuppressive tumour microenvironments (TMEs), and limited CAR T-cell persistence, all of which substantially reduce therapeutic efficacy (Zhou et al., 2022).

One of the earliest clinical targets was HER2 (ERBB2). Despite promising preclinical data, a Phase I trial reported fatal cytokine-mediated pulmonary toxicity following infusion of HER2-directed CAR T cells. Low-level HER2 expression in normal lung tissue resulted in severe “on-target, off-tumour” toxicity, fundamentally reshaping CAR T-cell development by emphasising the need for rigorous antigen selection, cautious dose escalation, and comprehensive preclinical safety evaluation (Morgan et al., 2010). Clinical studies targeting carcinoembryonic antigen (CEA) in metastatic colorectal cancer demonstrated successful tumour trafficking and occasional partial responses but also induced inflammatory colitis due to physiological CEA expression in normal intestinal epithelium. Although toxicity was manageable, these findings reinforced the challenge of identifying tumour-specific antigens that minimise damage to healthy tissues (Zhang et al., 2017).

Mesothelin-directed CAR T cells have shown favourable safety profiles in mesothelioma, pancreatic cancer, and ovarian cancer but have produced mainly disease stabilisation rather than durable tumour regression. Limited persistence, antigen heterogeneity, and profound immunosuppression within the TME appear to be the principal barriers to sustained clinical benefit (Haas et al., 2019). Similarly, CAR T cells targeting epidermal growth factor receptor variant III (EGFRvIII) successfully infiltrated glioblastoma but failed to produce durable responses. Post-treatment analyses revealed loss of EGFRvIII expression together with increased infiltration of regulatory T cells, tumour-associated macrophages, and elevated PD-L1 expression, providing clear evidence of antigen escape and adaptive immune resistance (O'Rourke et al., 2017).

Other targets, including glypican-3 (GPC3), GD2, MUC1, prostate-specific membrane antigen (PSMA), Claudin 18.2, and B7-H3, have demonstrated encouraging safety and occasional objective responses in early-phase trials. However, durable complete remissions remain uncommon, indicating that antigen recognition alone is insufficient to overcome the biological complexity of solid tumours (Qin and Xu, 2022). Collectively, these studies have identified several recurring barriers. Antigen heterogeneity promotes immune escape through selective expansion of antigen-negative tumour cells, while dense extracellular matrix, abnormal tumour vasculature, and cancer-associated fibroblasts restrict CAR T-cell infiltration (Rosa et al., 2026). In addition, immunosuppressive mediators such as transforming growth factor-β, interleukin-10, adenosine, indoleamine 2,3-dioxygenase, regulatory T cells, and myeloid-derived suppressor cells inhibit CAR T-cell proliferation and cytotoxicity (Pu et al., 2023). Persistent antigen stimulation also promotes T-cell exhaustion through increased expression of inhibitory receptors, including PD-1, TIM-3, and LAG-3 (Tang et al., 2022).

These lessons have driven the development of next-generation CAR T-cell strategies, including dual-target and logic-gated CARs, armoured CAR T cells that resist immunosuppression, enhanced trafficking through chemokine receptor engineering, and combination therapies with checkpoint inhibitors, radiotherapy, targeted agents, or oncolytic viruses (Bagley et al., 2024; Du et al., 2025). Although early clinical efficacy has been modest, these studies have defined the principal biological barriers that must be overcome to achieve durable therapeutic success in solid tumours (Smirnov et al., 2025). Figure 4 displays clinical trials of CAR-T cell therapy of solid tumours.

**FIGURE 4:**
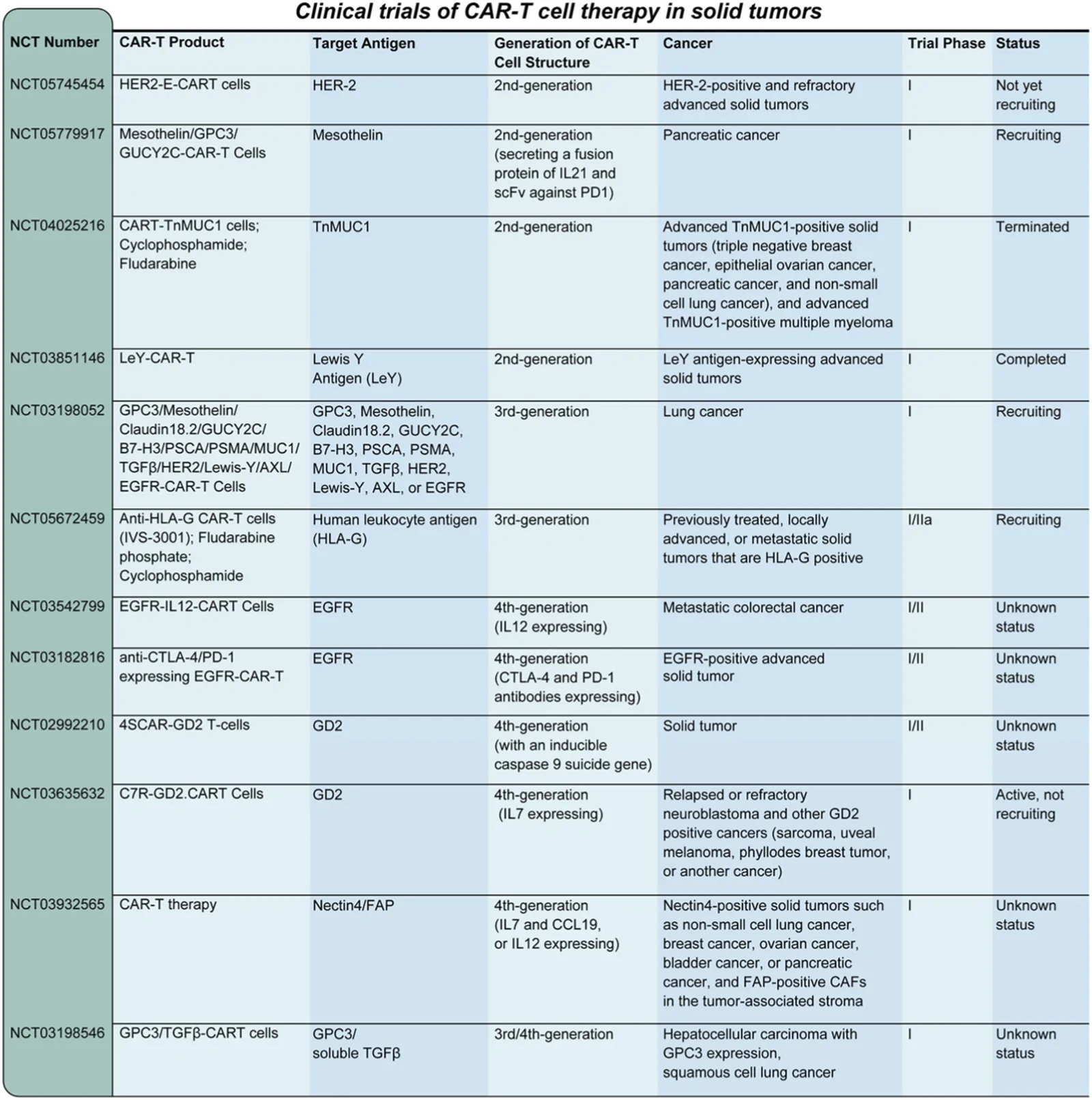
Clinical trials of CAR-T cell therapy of solid tumours. Modified from (Kuznetsova et al., 2024).

## 14 Biomarkers and predictive models in CAR-T-cell therapy-associated toxicity

### 14.1 Circulating biomarkers for early toxicity prediction

The identification of reliable biomarkers capable of predicting cytokine release syndrome (CRS) and immune effector cell-associated neurotoxicity syndrome (ICANS) before clinical deterioration remains an important objective in CAR-T-cell therapy. Routine inflammatory markers, including ferritin and C-reactive protein (CRP), are among the most extensively studied because they reflect systemic inflammation and macrophage activation. Although neither marker alone provides sufficient predictive accuracy, persistently elevated concentrations have been associated with severe CRS and poorer clinical outcomes (Li et al., 2024e). Subsequent studies have confirmed that combining ferritin and CRP with additional inflammatory mediators improves risk stratification for severe CRS (Wei Z. et al., 2023; Su et al., 2024). Recent evidence also highlights the value of integrating multiple blood biomarkers rather than relying on a single laboratory parameter for toxicity prediction as shown in Figure 5 (Weinkove and Jarvis, 2026).

**FIGURE 5:**
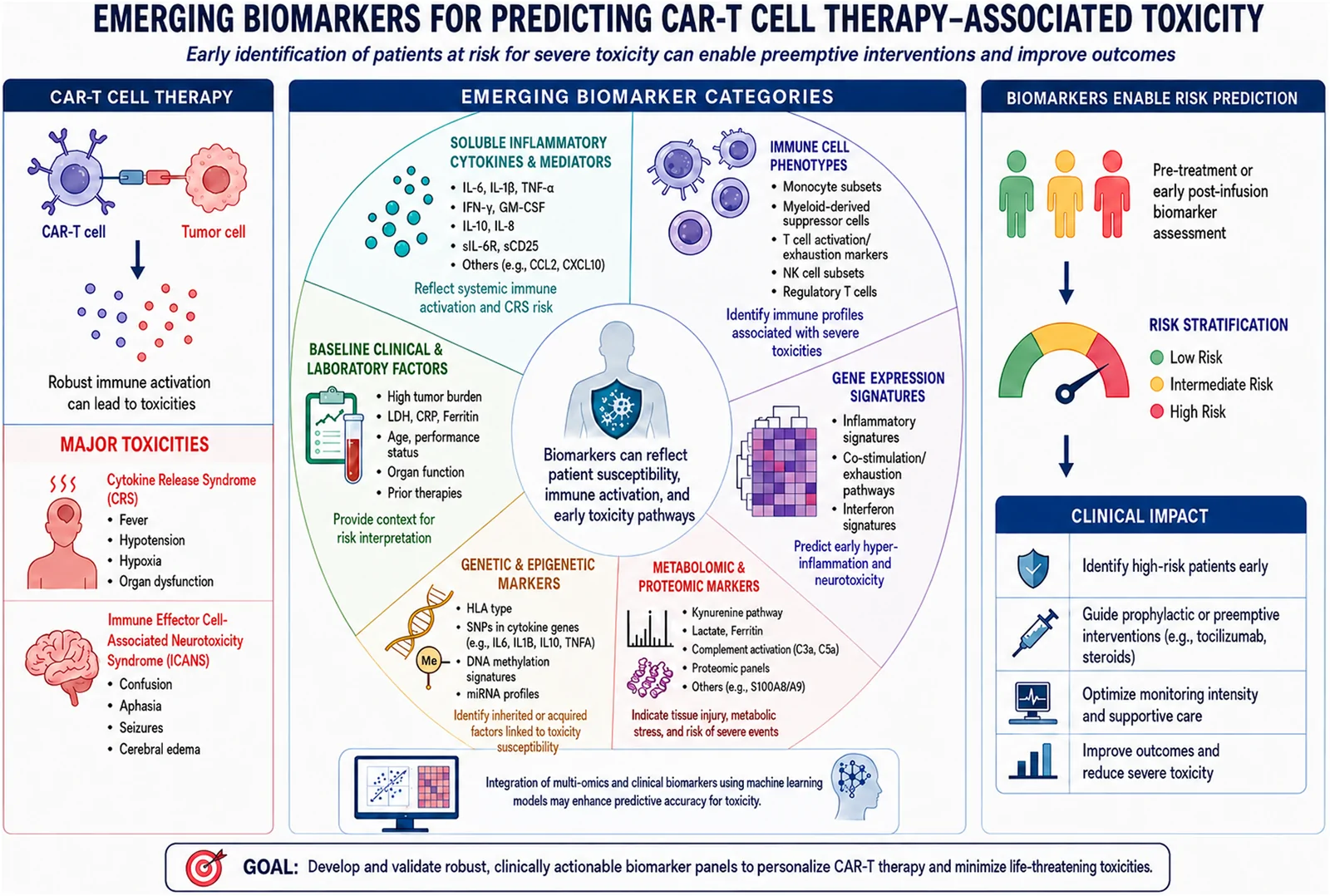
Emerging biomarkers for predicting CAR-T cell therapy-associated toxicity. This schematic summarizes the principal classes of biomarkers currently under investigation for the early prediction and risk stratification of adverse events following chimeric antigen receptor T-cell (CAR-T) therapy. The left panel illustrates the interaction between CAR-T cells and tumor cells, emphasizing that robust immune activation can trigger major treatment-related toxicities, including cytokine release syndrome (CRS) and immune effector cell-associated neurotoxicity syndrome (ICANS). The central panel categorizes emerging biomarkers into soluble inflammatory cytokines and mediators (e.g., IL-6, IL-1β, IFN-γ, GM-CSF, IL-10, and soluble IL-6 receptor), baseline clinical and laboratory parameters (including tumor burden, C-reactive protein, ferritin, lactate dehydrogenase, age, and prior therapies), immune cell phenotypes, gene expression signatures, genetic and epigenetic markers, and metabolic and proteomic indicators. Together, these biomarkers reflect host susceptibility, immune activation, inflammatory status, and tissue injury, providing complementary information on the likelihood and severity of treatment-associated toxicities. The right panel highlights their clinical applications, including pre-treatment and early post-infusion risk assessment, patient stratification into low-, intermediate-, and high-risk categories, implementation of pre-emptive interventions such as cytokine-directed therapies and corticosteroids, optimization of monitoring intensity, and personalized supportive care. Integration of multi-omics datasets with clinical variables through machine learning and artificial intelligence is expected to improve predictive accuracy and facilitate the development of validated biomarker panels capable of enhancing the safety and precision of CAR-T cell therapy.

### 14.2 Expanding biomarker classes and predictive technologies

Current research is expanding beyond conventional inflammatory markers to include biomarkers of endothelial dysfunction, haemostatic activation, immune-cell activation, and tumour biology. Endothelial injury markers such as angiopoietin-2, von Willebrand factor, thrombomodulin, D-dimer, and fibrinogen have been associated with severe CRS, ICANS, and coagulation abnormalities, highlighting the importance of vascular injury in CAR-T-cell toxicities (Moreno-Castaño et al., 2025; Demosthenous et al., 2025). In parallel, integrated biomarker panels are increasingly being combined with computational approaches to improve risk prediction. Early studies using gradient-boosting algorithms and other machine-learning models have demonstrated promising accuracy for identifying patients at risk of severe ICANS and high-grade CRS before clinical deterioration (Shahid and Curran, 2025; Huang et al., 2024; Yu et al., 2025). Additional biomarker classes, including cytokine signatures, immune-cell phenotyping, circulating tumour DNA, metabolomics, and single-cell sequencing, are expected to further refine individualized monitoring and therapeutic decision-making (Pu et al., 2023). Furthermore, soluble interleukin-2 receptor α (sIL-2Rα), a marker of T-cell activation, has emerged as a promising complementary biomarker that may enhance the predictive value of inflammatory markers such as ferritin and CRP when incorporated into multiparametric models (Oportus et al., 2025).

### 14.3 Machine learning for biomarker-based risk prediction

Machine-learning approaches are increasingly being applied to integrate clinical variables with laboratory biomarkers for individualized prediction of CAR-T-cell toxicities. Gradient-boosting algorithms and other artificial intelligence models have demonstrated encouraging performance in predicting severe ICANS and high-grade CRS before clinical onset (Huang et al., 2024; Yu et al., 2025). As larger multicentre datasets become available, these computational models may facilitate real-time risk stratification and support precision management of CAR-T-cell recipients (Yu et al., 2025).

## 15 Cost, accessibility, and emerging strategies to improve the affordability of CAR-T cell therapy

Despite the remarkable clinical success of CAR-T cell therapy in haematological malignancies, its widespread implementation remains constrained by substantial economic and logistical challenges. Currently approved autologous CAR-T products are among the most expensive cancer therapies available, with total treatment costs frequently exceeding USD 400,000–500,000 per patient when manufacturing, hospitalization, lymphodepleting chemotherapy, toxicity management, and supportive care are considered (Gonçalves, 2025; Gye et al., 2024; Dorneles et al., 2024; Gonçalves, 2025; Cavallo et al., 2024).

Additional indirect costs, including prolonged inpatient monitoring, intensive care admission for severe cytokine release syndrome (CRS) or immune effector cell-associated neurotoxicity syndrome (ICANS), and post-treatment follow-up, further increase the overall financial burden on healthcare systems (Gonçalves, 2025; Gye et al., 2024; Shahid and Curran, 2025). Consequently, access to CAR-T therapy remains largely restricted to specialised tertiary centres in high-income countries, creating significant disparities in global cancer care (Gonçalves, 2025; Ayala Ceja et al., 2024). The individualized manufacturing process of autologous CAR-T cells represents one of the principal drivers of cost (Ayala Ceja et al., 2024; Abdo et al., 2025; Shah et al., 2023; Khang et al., 2023).

Patient-derived T cells must be collected by leukapheresis, genetically modified under Good Manufacturing Practice (GMP) conditions, expanded *ex vivo*, subjected to rigorous quality control testing, cryopreserved, and transported back to the treating centre (Ayala Ceja et al., 2024; Abdo et al., 2025). This complex production workflow typically requires two to 4 weeks, during which patients with rapidly progressive disease may experience clinical deterioration or become ineligible for treatment (Abdo et al., 2025; Shah et al., 2023; Khang et al., 2023).

Manufacturing failures resulting from poor T-cell quality, prior chemotherapy exposure, or inadequate cell expansion further increase costs and delay treatment (Abdo et al., 2025; Chen and van den Brink, 2024). To address these limitations, considerable research has focused on the development of allogeneic “off-the-shelf” CAR-T cells, generated from healthy donors rather than individual patients (Chen and van den Brink, 2024; Mohty and Lazaryan, 2024; Lonez and Breman, 2024; Khadiullina et al., 2026; Mansoori et al., 2024). These products can be manufactured in advance, cryopreserved, and made immediately available when required, substantially reducing production time and potentially lowering manufacturing costs through large-scale production (Chen and van den Brink, 2024; Mohty and Lazaryan, 2024; Khadiullina et al., 2026).

Advances in genome editing technologies, including CRISPR/Cas9, TALENs, and zinc-finger nucleases, have enabled disruption of endogenous T-cell receptors and human leukocyte antigen (HLA) molecules, thereby reducing the risks of graft-versus-host disease (GVHD) and host immune rejection (Mohty and Lazaryan, 2024; Lonez and Breman, 2024; Khadiullina et al., 2026; Mansoori et al., 2024; Diorio et al., 2025). Although several allogeneic CAR-T products have entered early-phase clinical trials with encouraging preliminary efficacy and acceptable safety profiles, challenges including limited persistence, immune-mediated rejection, and optimal gene-editing strategies remain active areas of investigation (Mohty and Lazaryan, 2024; Lonez and Breman, 2024; Mansoori et al., 2024).

Another promising strategy for improving cost-effectiveness involves combining CAR-T cell therapy with targeted agents capable of enhancing antitumour activity or overcoming resistance (Du et al., 2025; Rosa et al., 2026). Small-molecule inhibitors, including Bruton tyrosine kinase (BTK) inhibitors, BCL-2 inhibitors, PI3K inhibitors, and tyrosine kinase inhibitors, may improve CAR-T cell expansion, persistence, or tumour susceptibility while potentially allowing lower CAR-T cell doses (Du et al., 2025; Rosa et al., 2026). Similarly, immune checkpoint inhibitors targeting PD-1/PD-L1 or CTLA-4 have demonstrated the ability to reverse CAR-T cell exhaustion and restore antitumour function in selected patients (Rosa et al., 2026; Qin and Xu, 2022).

Technological innovations aimed at simplifying manufacturing are also expected to reduce costs (Ayala Ceja et al., 2024; Abdo et al., 2025; Weltin et al., 2026). Automated closed-system manufacturing platforms, non-viral gene transfer methods such as transposon-based systems (Sleeping Beauty and PiggyBac), and CRISPR-mediated gene insertion have shown promise as less expensive alternatives to conventional viral vector production (Abdo et al., 2025; Weltin et al., 2026). Furthermore, decentralised manufacturing models and point-of-care production facilities may shorten manufacturing times, decrease transportation costs, and improve accessibility, particularly in regions lacking commercial production infrastructure (Ayala Ceja et al., 2024; Weltin et al., 2026).

Alternative immune cell platforms may further expand accessibility. Natural killer (NK) cell-based CAR therapies, macrophage CARs (CAR-M), invariant natural killer T (iNKT) cells, and γδ T-cell CARs can potentially be produced as universal donor products with lower risks of GVHD, allowing large-scale manufacturing and reduced production costs (Khadiullina et al., 2026; Diorio et al., 2025; Smirnov et al., 2025). While these approaches remain largely investigational, they represent attractive options for developing more affordable and broadly accessible cellular immunotherapies (Khadiullina et al., 2026; Smirnov et al., 2025). Health-economic analyses increasingly suggest that although CAR-T therapy involves exceptionally high upfront costs, durable complete remissions and long-term survival may render treatment cost-effective in selected patient populations by reducing subsequent hospitalisations, salvage chemotherapy, and stem cell transplantation (Gonçalves, 2025; Dorneles et al., 2024; Gonçalves, 2025; Gye et al., 2024; June and Sadelain, 2018). Nevertheless, affordability remains highly dependent on national healthcare reimbursement policies, manufacturing efficiency, and patient selection (Gonçalves, 2025; Gye et al., 2024).

Future reductions in manufacturing complexity, expansion of allogeneic products, integration of combination therapies, and improvements in production technologies are expected to play pivotal roles in making CAR-T therapy more accessible worldwide (Ayala Ceja et al., 2024; Chen and van den Brink, 2024; Weltin et al., 2026). Overall, improving affordability and accessibility is now recognised as one of the major priorities in next-generation CAR-T development (Gonçalves, 2025; Ayala Ceja et al., 2024; Chen and van den Brink, 2024). Advances in universal donor platforms, genome editing, automated manufacturing, and rational combination strategies have the potential to substantially reduce costs while maintaining therapeutic efficacy, thereby enabling broader global implementation of cellular immunotherapy (Chen and van den Brink, 2024; Mohty and Lazaryan, 2024; Lonez and Breman, 2024; Khadiullina et al., 2026; Mansoori et al., 2024; Diorio et al., 2025; Du et al., 2025; Rosa et al., 2026; Qin and Xu, 2022; Weltin et al., 2026).

## 16 Challenges for broader clinical translation of CAR-T-cell therapy

Despite the transformative clinical success of CAR-T-cell therapy in selected haematological malignancies, its broader implementation across different cancer types and healthcare systems remains constrained by multiple biological, technological, regulatory, and economic challenges. Although remarkable remission rates have been achieved in B-cell malignancies, these outcomes have not been consistently replicated in solid tumours, highlighting the complexity of translating experimental advances into routine clinical practice (Albelda, 2024; Li et al., 2025).

One of the principal biological barriers is tumour heterogeneity. Unlike B-cell malignancies, solid tumours frequently exhibit variable antigen density, spatial heterogeneity, antigen modulation, and antigen loss during treatment. Consequently, CAR-T cells may eliminate only a subset of malignant cells while permitting antigen-negative clones to survive and expand. Furthermore, the tumour microenvironment remains profoundly immunosuppressive, characterised by dense extracellular matrix deposition, abnormal tumour vasculature, hypoxia, nutrient deprivation, tumour-associated macrophages, regulatory T cells, myeloid-derived suppressor cells, inhibitory cytokines such as transforming growth factor-β, and checkpoint molecules including PD-1/PD-L1. Together, these mechanisms impair CAR-T-cell trafficking, persistence, proliferation, and cytotoxic function despite adequate antigen recognition (Albelda, 2024; Li et al., 2025).

Manufacturing continues to represent another major obstacle. Current autologous CAR-T-cell products require individualised manufacturing from patient-derived lymphocytes, resulting in prolonged vein-to-vein intervals, variable starting material quality, manufacturing failures, and substantial production costs. Patients with rapidly progressive disease may deteriorate clinically before treatment becomes available, while previous chemotherapy may compromise T-cell fitness and ultimately reduce product efficacy. Although automation, closed-system manufacturing, point-of-care production, and non-viral engineering platforms have shown considerable promise, these technologies require further validation before they can be widely implemented (Dias et al., 2024; Ayala Ceja et al., 2024; Urbano-Ispizua et al., 2024).

The development of allogeneic “off-the-shelf” CAR-cell products has generated considerable enthusiasm because donor-derived cells could substantially reduce manufacturing delays and improve global accessibility. Nevertheless, host immune rejection, limited cellular persistence, graft-versus-host disease, gene-editing safety, chromosomal alterations, and regulatory concerns remain important barriers to routine clinical use. Consequently, the theoretical advantages of allogeneic CAR products have not yet translated into universal clinical superiority over autologous therapies (Diorio et al., 2025).

Treatment-related toxicity continues to limit broader implementation. Cytokine release syndrome, immune effector cell-associated neurotoxicity syndrome, prolonged cytopenias, opportunistic infections, hypogammaglobulinaemia, endothelial dysfunction, and delayed neurological complications frequently require specialised multidisciplinary care available only in highly experienced tertiary centres. The identification of robust predictive biomarkers, together with controllable CAR systems, suicide switches, logic-gated receptors, affinity-tuned constructs, and cytokine-regulated CAR platforms, may substantially improve the therapeutic index while preserving antitumour activity (Brudno and Kochenderfer, 2024).

Regulatory approval and health economics represent additional but often underappreciated challenges. CAR-T-cell products require highly complex manufacturing oversight, long-term pharmacovigilance, standardised potency assays, and extensive post-marketing surveillance to ensure product consistency and patient safety. At the same time, the exceptionally high cost of treatment—including cell manufacturing, hospitalisation, toxicity management, and prolonged follow-up—places considerable pressure on healthcare systems and limits equitable access, particularly in low- and middle-income countries. Demonstrating long-term cost-effectiveness alongside durable clinical benefit will therefore become increasingly important for reimbursement and widespread adoption.

Future progress will likely depend on the integration of multiple emerging technologies rather than improvements in receptor design alone. Artificial intelligence and machine-learning algorithms are increasingly being applied to optimise antigen selection, predict toxicity, improve manufacturing quality control, and personalise CAR design. Likewise, advances in CRISPR/Cas9 genome editing, synthetic biology, multiplex gene engineering, induced pluripotent stem cell-derived immune cells, universal CAR platforms, and combination strategies incorporating checkpoint inhibition, cytokines, or targeted therapies may significantly enhance efficacy while improving safety and scalability. However, these innovations require rigorous clinical validation before they can be incorporated into standard clinical practice (Li et al., 2025; Ayala Ceja et al., 2024).

An additional challenge that is likely to influence the future adoption of CAR-T-cell therapy is the need for greater standardisation across manufacturing, regulatory oversight, and patient selection. Considerable variability currently exists in manufacturing platforms, release criteria, potency assays, and institutional experience, making direct comparisons between clinical studies difficult and complicating global implementation. Furthermore, the emergence of precision medicine approaches has highlighted the importance of identifying predictive biomarkers capable of selecting patients most likely to benefit from CAR-based therapies while minimising severe toxicities. Advances in multi-omics technologies, artificial intelligence, and digital pathology are expected to facilitate patient stratification, optimise antigen selection, and improve treatment individualisation. At the same time, synthetic biology is enabling the development of programmable CAR circuits, Boolean logic-gated receptors, and self-regulating cellular therapies with improved tumour specificity and safety. Continued progress in non-viral gene transfer systems, CRISPR-mediated genome editing, and induced pluripotent stem cell-derived immune products may further reduce manufacturing costs and improve scalability. Collectively, these technological advances suggest that the next-generation of CAR therapies will depend as much on engineering innovation and precision patient selection as on improvements in receptor biology (Depil et al., 2024).

The future clinical success of CAR-T-cell therapy will depend on overcoming challenges that extend well beyond receptor engineering. Continued progress requires integrated advances in antigen discovery, manufacturing technologies, genome editing, toxicity prediction, regulatory science, and health economics. Automation, decentralised manufacturing, and allogeneic cell platforms may improve treatment accessibility; however, these innovations must demonstrate consistent product quality, long-term safety, and cost-effectiveness before widespread implementation can be achieved. Furthermore, the integration of artificial intelligence, multi-omics technologies, and synthetic biology is expected to facilitate patient selection, optimise CAR design, and enable more precise prediction of treatment efficacy and toxicity. At the same time, harmonised international regulatory frameworks and equitable reimbursement strategies will be essential to expand access beyond highly specialised centres. Consequently, the future of CAR-based immunotherapy will depend on coordinated scientific, technological, clinical, regulatory, and economic innovation rather than improvements in receptor design alone (Li et al., 2025; Dias et al., 2024; Ayala Ceja et al., 2024; Brudno and Kochenderfer, 2024).

Beyond technological innovation, the long-term success of CAR-T-cell therapy will depend on the ability to integrate scientific advances into sustainable clinical practice. As indications expand beyond highly selected patients, greater emphasis must be placed on generating robust real-world evidence to complement clinical trial data and better define long-term effectiveness, late toxicities, and patient-reported outcomes across diverse populations. Future implementation will also require international collaboration to harmonise manufacturing standards, outcome reporting, and regulatory pathways, thereby facilitating broader access to high-quality cellular therapies. Moreover, personalised treatment algorithms that integrate tumour genomics, immune profiling, and dynamic biomarkers may enable more precise selection of both patients and CAR constructs, reducing unnecessary toxicity while maximising therapeutic benefit. These developments will shift CAR-T-cell therapy from a predominantly experimental and highly specialised intervention toward a more adaptable precision medicine platform. Ultimately, the greatest challenge is no longer demonstrating that CAR-T cells can eradicate cancer, but ensuring that these therapies can be delivered safely, consistently, equitably, and sustainably to the patients most likely to benefit from them (Litvinova et al., 2024; Ge et al., 2024; Sainatham et al., 2024; Cavallo et al., 2024; Alipour-Haris et al., 2024; Ge et al., 2026).

Overall, the future success of CAR-T-cell therapy will depend not only on overcoming tumour biology but also on addressing manufacturing efficiency, affordability, regulatory harmonisation, equitable global access, and long-term safety. Only through coordinated advances across these interconnected domains can CAR-based immunotherapy evolve from a highly specialised treatment into a broadly accessible platform for precision cancer medicine.

## 17 Switchable and universal CAR-T systems: future perspectives

Switchable and universal CAR-T-cell platforms represent an emerging strategy to improve the safety, adaptability, and clinical versatility of adoptive cellular immunotherapy. Unlike conventional CAR-T cells, which possess fixed antigen specificity following infusion, switchable CAR systems separate tumour recognition from T-cell activation through the use of adaptor molecules or soluble targeting bridges that can be administered, modified, or withdrawn according to clinical need. This modular design permits temporal regulation of CAR-T-cell activity, dose adjustment, sequential targeting of different tumour-associated antigens, and rapid cessation of immune activation in the event of severe toxicity (Brudno et al., 2018). Universal CAR platforms may also reduce tumour escape by allowing retargeting without manufacturing new cellular products, potentially extending therapeutic durability in heterogeneous malignancies. Nevertheless, these systems introduce additional biological and logistical challenges, including dependence on repeated administration of adaptor molecules, potential immunogenicity, pharmacokinetic variability, increased manufacturing complexity, and higher treatment costs. Furthermore, clinical experience remains limited, with most switchable CAR platforms currently evaluated in early-phase clinical trials or preclinical studies. Their long-term persistence, efficacy in solid tumours, and capacity to reduce cytokine release syndrome or immune effector cell-associated neurotoxicity syndrome have yet to be demonstrated conclusively. Future research should therefore prioritise optimisation of adaptor molecule design, evaluation of long-term safety, integration with logic-gated and allogeneic CAR technologies, and comparative clinical trials to determine whether switchable systems provide meaningful advantages over established second-generation CAR-T-cell therapies (Brudno et al., 2018; Hort et al., 2022; Portale and Di Mitri, 2023; Yu, 2023; Zhou et al., 2023) (Figure 6).

**FIGURE 6:**
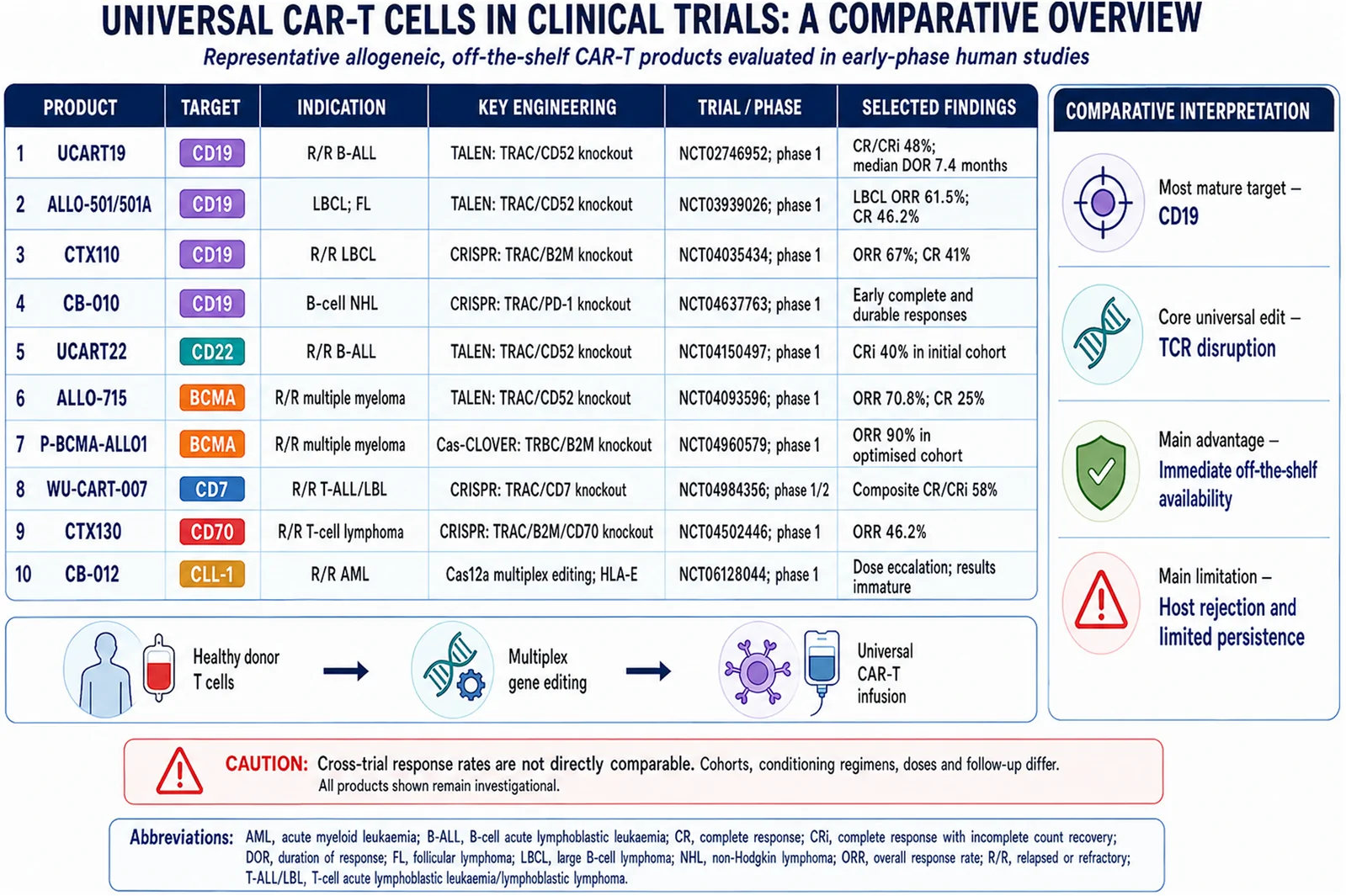
Clinical landscape and comparative characteristics of universal chimeric antigen receptor T-cell therapies. Representative allogeneic, off-the-shelf CAR-T products undergoing early-phase clinical evaluation are compared according to their tumour-associated target, indication, genome-engineering strategy, trial phase and selected efficacy findings. Most platforms use healthy-donor T cells with disruption of the endogenous T-cell receptor to mitigate graft-versus-host disease, frequently accompanied by additional edits designed to prevent fratricide, reduce host-mediated rejection or enhance functional persistence. CD19-directed products currently have the most mature clinical evidence, whereas BCMA-, CD7-, CD70- and CLL-1-directed approaches extend this strategy to multiple myeloma, T-cell malignancies and acute myeloid leukaemia. Although universal CAR-T cells offer immediate availability and scalable manufacture, limited *in-vivo* persistence, host-versus-graft rejection and the toxicity associated with lymphodepleting regimens remain important barriers. Reported response rates should not be compared directly because the studies differ in patient populations, conditioning regimens, administered doses, response-evaluable cohorts and duration of follow-up. All products shown remain investigational. AML, acute myeloid leukaemia; B-ALL, B-cell acute lymphoblastic leukaemia; CR, complete response; CRi, complete response with incomplete haematological recovery; DOR, duration of response; FL, follicular lymphoma; LBCL, large B-cell lymphoma; NHL, non-Hodgkin lymphoma; ORR, overall response rate; R/R, relapsed or refractory; T-ALL/LBL, T-cell acute lymphoblastic leukaemia/lymphoblastic lymphoma.

## 18 CLDN18.2-directed CAR-T therapy: a landmark advance in solid-tumour immunotherapy

CLDN18.2-directed chimeric antigen receptor T-cell therapy represents a landmark transition in cancer immunotherapy. CAR-T treatments transformed several B-cell malignancies, yet comparable success in solid tumours remained elusive because malignant cells often lack a uniformly expressed, tumour-selective surface antigen. Dense stroma, impaired T-cell trafficking, antigen heterogeneity and an immunosuppressive tumour microenvironment also restrict cellular penetration, persistence and cytotoxicity. Satricabtagene autoleucel (satri-cel; formerly CT041) provided early clinical proof that these barriers could be overcome, at least partly, through careful antigen selection and biomarker-guided treatment (Qi et al., 2022) (Figure 7).

**FIGURE 7:**
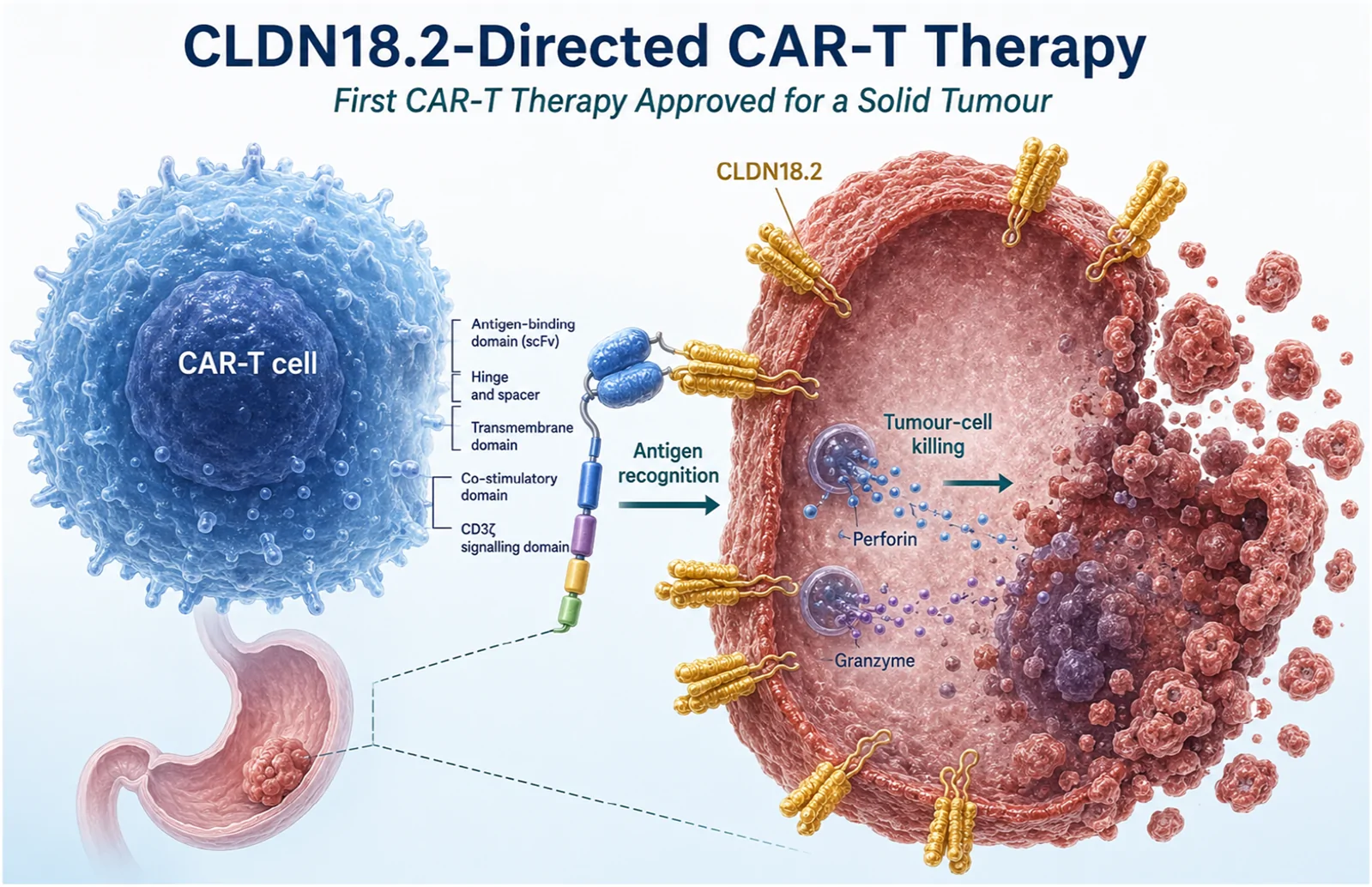
CLDN18.2-directed CAR-T therapy: a landmark advance in solid-tumour immunotherapy. The illustration summarises the biological rationale and therapeutic action of chimeric antigen receptor (CAR) T cells targeting claudin 18 isoform 2 (CLDN18.2).

CLDN18.2 is a gastric-lineage tight-junction protein. In healthy tissue, it is largely confined to differentiated gastric mucosal cells, where its epitopes are normally concealed within intercellular junctions. Malignant transformation disrupts epithelial polarity, exposing CLDN18.2 on the cancer-cell surface while retaining expression in many gastric and gastro-oesophageal junction adenocarcinomas. This restricted distribution and tumour-associated accessibility make CLDN18.2 an attractive therapeutic target. Patient-derived T lymphocytes are genetically engineered to express a receptor that binds CLDN18.2 independently of major histocompatibility complex presentation. Antigen engagement activates intracellular co-stimulatory and CD3ζ signalling, prompting CAR-T-cell expansion, cytokine production and perforin- and granzyme-mediated tumour-cell death.

The pivotal randomised phase II CT041-ST-01 trial compared satri-cel with physician's-choice treatment in heavily pretreated patients with CLDN18.2-positive advanced gastric or gastro-oesophageal junction cancer. Satri-cel significantly prolonged median progression-free survival from 1.77 to 3.25 months (hazard ratio 0.37), establishing the first positive randomised evidence for CAR-T therapy in a solid tumour (Qi et al., 2025). In June 2026, China’s National Medical Products Administration approved satri-cel for eligible CLDN18.2-positive, HER2-negative advanced gastric or gastro-oesophageal junction adenocarcinoma after failure of at least two previous systemic treatment lines. It therefore became the first regulator-approved CAR-T-cell product worldwide specifically indicated for a solid tumour (Mullard, 2026).

This achievement should be interpreted as proof of principle rather than a universal solution. Responses remain influenced by antigen density, intratumoural heterogeneity, manufacturing time and the hostile tumour microenvironment. Treatment requires lymphodepletion and specialist monitoring for cytokine-release syndrome, cytopenias, infection and possible on-target gastric toxicity. Moreover, grade 3 or worse treatment-emergent adverse events were frequent in the pivotal trial, although cytokine-release syndrome was predominantly low grade. Nevertheless, the approval changes the conceptual boundaries of cellular therapy. It demonstrates that rational target selection can convert a previously resistant solid malignancy into a clinically actionable CAR-T indication and provides a platform for next-generation strategies incorporating dual targeting, improved trafficking, controlled activation and resistance to immunosuppressive signals.

## 19 Conclusion

CAR-T therapy for solid tumors is in its early clinical development stages, but preclinical studies and early-phase trials have shown promise. CAR-T cells targeting antigens like HER2, EGFRvIII, and mesothelin have demonstrated potential in treating breast cancer, glioblastoma, and mesothelioma, respectively. Ongoing research aims to refine these strategies, with the hope that CAR-T therapy will eventually offer effective treatment options for solid tumors, particularly for cancers that are difficult to treat. Despite significant challenges, advancements in target selection, CAR design, and combination therapies are paving the way for CAR-T cell therapy to become a powerful tool in oncology.

In studying CAR-T cell binding to antigens, conventional methods such as flow cytometry, ELISA, and immunohistochemistry are widely used due to their established protocols and accessibility. While these techniques provide valuable insights, they may lack the sensitivity and throughput required for more detailed analyses. Advanced methods like surface plasmon resonance (SPR), single-cell RNA sequencing (scRNA-seq), and CRISPR screening offer higher resolution data, enabling deeper understanding of CAR-T cell interactions with target antigens.

NK CAR cells, combining the innate cytotoxicity of NK cells with CAR technology, represent a promising advancement in cancer immunotherapy, potentially targeting a broad range of tumor cells with minimal adverse effects. Ongoing research and clinical trials are crucial to overcoming challenges in their development and expanding their clinical application. Universal CAR-T cells offer a transformative approach by overcoming the limitations of patient-specific therapies. Using TCR knockout T cells, NKT cells, and double-negative T cells, these universal CAR-T cells promise broader applicability, reduced risk of GVHD, and lower production costs. As research advances, universal CAR-T cells could revolutionise cancer treatment by making it more effective, accessible, and scalable for a wider patient population.

CAR-T cell therapy has emerged as one of the most significant advances in cancer immunotherapy, demonstrating remarkable clinical efficacy in relapsed and refractory haematological malignancies. However, despite its transformative impact, several scientific, clinical, and economic challenges continue to limit its broader application. This review highlights that overcoming antigen escape, tumour heterogeneity, and the immunosuppressive tumour microenvironment remains essential for extending the benefits of CAR-T therapy to patients with solid tumours. Comparative evaluation of multi-target strategies indicates that bispecific, tandem, and pooled CAR-T cell approaches each offer distinct advantages and limitations, with no single platform currently providing an optimal solution across all tumour types.

The review further demonstrates that innovations such as γδ CAR-T cells, allogeneic “off-the-shelf” CAR-T products, genome editing technologies, and combination therapies have the potential to improve therapeutic efficacy while addressing manufacturing constraints and treatment accessibility. Nevertheless, these emerging approaches require further optimisation to overcome challenges related to limited cellular persistence, immune rejection, large-scale production, and long-term safety. Likewise, advances in automated manufacturing and non-viral gene engineering may reduce production costs and increase global access to these therapies, although robust health-economic evaluations remain necessary.

Management of treatment-related toxicities also continues to evolve. While IL-6 remains central to the pathogenesis of cytokine release syndrome, accumulating evidence supports the incorporation of additional biomarkers, including ferritin, C-reactive protein, soluble interleukin-2 receptor alpha, and multiparametric cytokine profiles, to improve early risk stratification and guide personalised clinical management. The integration of biomarker panels with machine learning and other predictive models represents a promising direction for enhancing patient safety.

Overall, the future success of CAR-T cell therapy will depend on multidisciplinary advances in cellular engineering, biomarker discovery, manufacturing technologies, and clinical trial design. Continued translational research and carefully conducted clinical studies are essential to develop safer, more effective, and more accessible CAR-T therapies that can be successfully implemented across a wider range of malignancies and healthcare settings worldwide.

Beyond continued improvements in receptor engineering, the successful future of CAR-T-cell therapy will depend on overcoming the practical challenges that currently limit its widespread clinical implementation. Although autologous CAR-T-cell products have demonstrated remarkable efficacy in selected haematological malignancies, high manufacturing costs, prolonged production times, specialised infrastructure requirements, and complex reimbursement pathways continue to restrict equitable patient access, particularly in low- and middle-income countries. Advances in allogeneic “off-the-shelf” CAR-T-cell platforms, automated manufacturing technologies, genome editing, and standardised production processes offer promising opportunities to improve scalability and reduce treatment costs. However, these innovations must be accompanied by rigorous evaluation of long-term safety, persistence, cost-effectiveness, and regulatory harmonisation before they can achieve broad clinical adoption. Greater international collaboration, health technology assessment, and sustainable reimbursement strategies will be essential to ensure equitable global access to these transformative therapies.

Future progress will also depend on integrating precision medicine with advances in synthetic biology, computational modelling, and artificial intelligence. The identification of predictive biomarkers capable of stratifying patients according to treatment response and the risk of severe toxicities, including cytokine release syndrome and immune effector cell-associated neurotoxicity syndrome, may enable more individualised treatment selection and earlier therapeutic intervention. Artificial intelligence and machine learning are expected to facilitate optimisation of CAR design, antigen selection, manufacturing quality control, and real-time analysis of multidimensional clinical and molecular datasets, thereby supporting personalised cellular therapies. Together with programmable CAR systems, logic-gated receptors, and controllable immune circuits, these technologies have the potential to improve therapeutic precision while reducing toxicity and treatment failure. Continued multidisciplinary collaboration between immunologists, bioengineers, computational scientists, clinicians, and regulatory authorities will be fundamental for translating these next-generation innovations into safe, effective, and globally accessible cancer immunotherapies.
