# Supplementary material for: The future directions of CAR-T Cell therapy: unlocking the potential of immunotherapy in cancer treatment
Source: Front Mol Med. 2026 Sep 15;6:1931159. doi: 10.3389/fmmed.2026.1931159 (PMC13620351; doi:10.3389/fmmed.2026.1931159)
Supplement: Supplementary file 1 [file Supplementaryfile1.docx]

### Recommended title

**Table 1. FDA-Approved Autologous CAR-T-Cell Products for Relapsed or Refractory B-Cell and Plasma-Cell Malignancies**

FDA approval status and indications verified as of 20 August 2026.

| **Generic name (brand)** | **Current manufacturer** | **Target and CAR design** | **Initial FDA approval** | **Principal current FDA-approved indications** | **Selected evidence** |
| --- | --- | --- | --- | --- | --- |
| **Tisagenlecleucel (Kymriah)** | Novartis Pharmaceuticals Corporation | **CD19**; second-generation CAR containing a 4-1BB co-stimulatory domain and CD3ζ activation domain; lentiviral gene transfer | 2017 | Patients ≤25 years with refractory B-cell precursor ALL or disease in second or later relapse; adults with R/R LBCL after ≥2 systemic treatment lines; adults with R/R FL after ≥2 lines | (FDA, 2025a; Maude et al., 2018; Schuster et al., 2019) |
| **Axicabtagene ciloleucel (Yescarta)** | Kite Pharma, a Gilead company | **CD19**; second-generation CAR containing a CD28 co-stimulatory domain and CD3ζ activation domain; γ-retroviral gene transfer | 2017 | Adults with LBCL refractory to first-line chemoimmunotherapy or relapsing within 12 months; R/R LBCL after ≥2 systemic treatment lines; R/R FL after ≥2 lines | (FDA, 2026a; Neelapu et al., 2017) |
| **Brexucabtagene autoleucel (Tecartus)** | Kite Pharma, a Gilead company | **CD19**; second-generation CD28–CD3ζ CAR; γ-retroviral gene transfer. The manufacturing process includes enrichment intended to reduce malignant B-cell contamination | 2020 | Adults with R/R MCL; adults with R/R B-cell precursor ALL | (FDA, 2026b; Wang et al., 2020) |
| **Lisocabtagene maraleucel (Breyanzi)** | Juno Therapeutics, a Bristol Myers Squibb company | **CD19**; second-generation 4-1BB–CD3ζ CAR; lentiviral gene transfer. Administered as separately manufactured CD4⁺ and CD8⁺ CAR-T-cell components at a defined composition | 2021 | Selected adults with R/R or early-relapsing LBCL; R/R CLL or SLL after ≥2 lines, including a BTK inhibitor and BCL-2 inhibitor; R/R FL after ≥2 lines; R/R MCL after ≥2 lines, including a BTK inhibitor; R/R MZL after ≥2 lines | (FDA, 2026c; Abramson et al., 2020; Kamdar et al., 2022) |
| **Idecabtagene vicleucel (Abecma)** | Celgene Corporation, a Bristol Myers Squibb company | **BCMA**; second-generation 4-1BB–CD3ζ CAR; lentiviral gene transfer | 2021 | Adults with R/R multiple myeloma after ≥2 previous treatment lines, including an immunomodulatory agent, a proteasome inhibitor and an anti-CD38 monoclonal antibody | (FDA, 2025b; Munshi et al., 2021; Rodriguez-Otero et al., 2023) |
| **Ciltacabtagene autoleucel (Carvykti)** | Janssen Biotech; developed and marketed with Legend Biotech | **BCMA**; second-generation CAR containing two BCMA-binding single-domain antibodies, a 4-1BB co-stimulatory domain and CD3ζ activation domain; lentiviral gene transfer | 2022 | Adults with R/R multiple myeloma after ≥1 previous treatment line that included a proteasome inhibitor and an immunomodulatory agent, and whose disease is refractory to lenalidomide | (FDA, 2025c; Berdeja et al., 2021; San-Miguel et al., 2023) |
| **Obecabtagene autoleucel (Aucatzyl)** | Autolus Limited | **CD19**; second-generation 4-1BB–CD3ζ CAR incorporating an intermediate-affinity, fast off-rate CD19-binding domain; lentiviral gene transfer | 2024 | Adults with R/R B-cell precursor ALL. The total dose is administered in two fractions, with the second dose guided by disease burden and toxicity following the first infusion | (FDA, 2026d; Roddie et al., 2024) |

**Abbreviations:** ALL, acute lymphoblastic leukaemia; BCMA, B-cell maturation antigen; BCL-2, B-cell lymphoma 2; BTK, Bruton tyrosine kinase; CLL, chronic lymphocytic leukaemia; FDA, United States Food and Drug Administration; FL, follicular lymphoma; LBCL, large B-cell lymphoma; MCL, mantle-cell lymphoma; MZL, marginal-zone lymphoma; R/R, relapsed or refractory; SLL, small lymphocytic lymphoma.

**Regulatory note:** In June 2025, the FDA eliminated the separate Risk Evaluation and Mitigation Strategies requirements for the approved CD19- and BCMA-directed autologous CAR-T products. However, prescribing information continues to include boxed warnings addressing serious risks such as cytokine-release syndrome, neurological toxicities and secondary haematological malignancies ([FDA, 2025d](https://www.fda.gov/news-events/press-announcements/fda-eliminates-risk-evaluation-and-mitigation-strategies-rems-autologous-chimeric-antigen-receptor)).

### Harvard reference list

Abramson, J.S., Palomba, M.L., Gordon, L.I., Lunning, M.A., Wang, M., Arnason, J. et al. (2020) ‘Lisocabtagene maraleucel for patients with relapsed or refractory large B-cell lymphomas: a phase 1, single-arm, multicentre study’, The Lancet, 396(10254), pp. 839–852. <https://doi.org/10.1016/S0140-6736(20)31366-0>

Berdeja, J.G., Madduri, D., Usmani, S.Z., Jakubowiak, A., Agha, M., Cohen, A.D. et al. (2021) ‘Ciltacabtagene autoleucel, a B-cell maturation antigen-directed chimeric antigen receptor T-cell therapy in patients with relapsed or refractory multiple myeloma’, The Lancet, 398(10297), pp. 314–324. <https://doi.org/10.1016/S0140-6736(21)00933-8>

FDA (2025a) ‘Kymriah (tisagenlecleucel)’. United States Food and Drug Administration. Available at: [FDA Kymriah product information](https://www.fda.gov/vaccines-blood-biologics/cellular-gene-therapy-products/kymriah) (Accessed: 20 August 2026).

FDA (2025b) ‘Abecma (idecabtagene vicleucel): prescribing information’. United States Food and Drug Administration. Available at: [FDA Abecma prescribing information](https://www.fda.gov/media/147055/download) (Accessed: 20 August 2026).

FDA (2025c) ‘Carvykti (ciltacabtagene autoleucel)’. United States Food and Drug Administration. Available at: [FDA Carvykti product information](https://www.fda.gov/vaccines-blood-biologics/cellular-gene-therapy-products/carvykti) (Accessed: 20 August 2026).

FDA (2025d) ‘FDA eliminates Risk Evaluation and Mitigation Strategies for autologous chimeric antigen receptor T-cell immunotherapies’. United States Food and Drug Administration, 27 June. Available at: [FDA safety announcement](https://www.fda.gov/news-events/press-announcements/fda-eliminates-risk-evaluation-and-mitigation-strategies-rems-autologous-chimeric-antigen-receptor) (Accessed: 20 August 2026).

FDA (2026a) ‘Yescarta (axicabtagene ciloleucel)’. United States Food and Drug Administration. Available at: [FDA Yescarta product information](https://www.fda.gov/vaccines-blood-biologics/cellular-gene-therapy-products/yescarta) (Accessed: 20 August 2026).

FDA (2026b) ‘Tecartus (brexucabtagene autoleucel)’. United States Food and Drug Administration. Available at: [FDA Tecartus product information](https://www.fda.gov/vaccines-blood-biologics/cellular-gene-therapy-products/tecartus) (Accessed: 20 August 2026).

FDA (2026c) ‘Breyanzi (lisocabtagene maraleucel)’. United States Food and Drug Administration. Available at: [FDA Breyanzi product information](https://www.fda.gov/vaccines-blood-biologics/cellular-gene-therapy-products/breyanzi) (Accessed: 20 August 2026).

FDA (2026d) ‘Aucatzyl (obecabtagene autoleucel)’. United States Food and Drug Administration. Available at: [FDA Aucatzyl product information](https://www.fda.gov/vaccines-blood-biologics/aucatzyl) (Accessed: 20 August 2026).

Kamdar, M., Solomon, S.R., Arnason, J., Johnston, P.B., Glass, B., Bachanova, V. et al. (2022) ‘Lisocabtagene maraleucel versus standard of care as second-line treatment in patients with relapsed or refractory large B-cell lymphoma: the TRANSFORM trial’, The Lancet, 399(10343), pp. 2294–2308. <https://doi.org/10.1016/S0140-6736(22)00662-6>

Maude, S.L., Laetsch, T.W., Buechner, J., Rives, S., Boyer, M., Bittencourt, H. et al. (2018) ‘Tisagenlecleucel in children and young adults with B-cell lymphoblastic leukaemia’, New England Journal of Medicine, 378(5), pp. 439–448. <https://doi.org/10.1056/NEJMoa1709866>

Munshi, N.C., Anderson, L.D., Shah, N., Madduri, D., Berdeja, J., Lonial, S. et al. (2021) ‘Idecabtagene vicleucel in relapsed and refractory multiple myeloma’, New England Journal of Medicine, 384(8), pp. 705–716. <https://doi.org/10.1056/NEJMoa2024850>

Neelapu, S.S., Locke, F.L., Bartlett, N.L., Lekakis, L.J., Miklos, D.B., Jacobson, C.A. et al. (2017) ‘Axicabtagene ciloleucel CAR T-cell therapy in refractory large B-cell lymphoma’, New England Journal of Medicine, 377(26), pp. 2531–2544. <https://doi.org/10.1056/NEJMoa1707447>

Rodriguez-Otero, P., Ailawadhi, S., Arnulf, B., Patel, K., Cavo, M., Nooka, A.K. et al. (2023) ‘Ide-cel or standard regimens in relapsed and refractory multiple myeloma’, New England Journal of Medicine, 388(11), pp. 1002–1014. <https://doi.org/10.1056/NEJMoa2213614>

Roddie, C., Sandhu, K.S., Tholouli, E., Logan, A.C., Shaughnessy, P., Barba, P. et al. (2024) ‘Obecabtagene autoleucel in adults with B-cell acute lymphoblastic leukaemia’, New England Journal of Medicine, 391(23), pp. 2219–2230. <https://doi.org/10.1056/NEJMoa2406526>

San-Miguel, J., Dhakal, B., Yong, K., Spencer, A., Anguille, S., Mateos, M.-V. et al. (2023) ‘Cilta-cel or standard care in lenalidomide-refractory multiple myeloma’, New England Journal of Medicine, 389(4), pp. 335–347. <https://doi.org/10.1056/NEJMoa2303379>

Schuster, S.J., Bishop, M.R., Tam, C.S., Waller, E.K., Borchmann, P., McGuirk, J.P. et al. (2019) ‘Tisagenlecleucel in adult relapsed or refractory diffuse large B-cell lymphoma’, New England Journal of Medicine, 380(1), pp. 45–56. <https://doi.org/10.1056/NEJMoa1804980>

Wang, M., Munoz, J., Goy, A., Locke, F.L., Jacobson, C.A., Hill, B.T. et al. (2020) ‘KTE-X19 CAR T-cell therapy in relapsed or refractory mantle-cell lymphoma’, New England Journal of Medicine, 382(14), pp. 1331–1342. <https://doi.org/10.1056/NEJMoa1914347>
