# Supplementary material for: The future directions of CAR-T Cell therapy: unlocking the potential of immunotherapy in cancer treatment
Source: Front Mol Med. 2026 Sep 15;6:1931159. doi: 10.3389/fmmed.2026.1931159 (PMC13620351; doi:10.3389/fmmed.2026.1931159)
Supplement: Supplementary file 2 [file Supplementaryfile2.docx]

**Table 2. End-to-End Manufacture, Clinical Administration and Biological Activity of CAR-T-Cell Products**

| **Phase** | **Step** | **Detailed process** | **Key considerations** | **Reference** |
| --- | --- | --- | --- | --- |
| Product manufacture | 1. Patient or donor selection | T cells are obtained from the patient for autologous therapy or from a healthy donor for allogeneic manufacture. Clinical eligibility, previous treatments, infection status and peripheral lymphocyte counts are assessed before collection. | Autologous products avoid graft-versus-host disease but may be affected by the patient’s disease and previous immunosuppressive treatment. Allogeneic products offer an immediately available source but require control of alloreactivity. | (Hartmann et al., 2017; Levine et al., 2017) |
| Product manufacture | 2. Leukapheresis | Peripheral blood mononuclear cells are collected by leukapheresis. The starting material contains T cells together with B cells, natural killer cells, monocytes and other circulating cells. | Collection must provide sufficient viable CD3⁺ cells. Identity, cell number, viability, microbial contamination and chain of custody should be documented. | (Levine et al., 2017; Ayala Ceja et al., 2024) |
| Product manufacture | 3. T-cell isolation and enrichment | CD3⁺ T cells, or defined CD4⁺ and CD8⁺ subsets, are isolated from the leukapheresis product using antibody-coated magnetic beads or other cell-selection systems. | The composition and differentiation state of the starting T-cell population can influence expansion, persistence and therapeutic activity. | (Turtle et al., 2016; Ayala Ceja et al., 2024) |
| Product manufacture | 4. T-cell activation | Isolated T cells are stimulated, commonly through CD3 and CD28, to induce activation, proliferation and susceptibility to genetic modification. | Excessive or prolonged stimulation can promote terminal differentiation and T-cell exhaustion. Activation conditions must therefore be carefully controlled. | (Levine et al., 2017; Ayala Ceja et al., 2024) |
| Product manufacture | 5. CAR design | The CAR generally contains an extracellular antigen-binding domain, usually a single-chain variable fragment; a hinge or spacer; a transmembrane domain; and intracellular activation and co-stimulatory domains. | The selection of antigen-binding, hinge, transmembrane and signalling elements affects specificity, activation strength, persistence and toxicity. | (Sadelain, Brentjens and Rivière, 2013) |
| Product manufacture | 6. Selection of CAR generation | First-generation CARs contain CD3ζ or another activation domain alone. Second-generation CARs add one co-stimulatory domain, commonly CD28 or 4-1BB. Third-generation CARs contain two co-stimulatory domains. | Second-generation CARs form the basis of most established clinical products. Third-generation constructs may enhance expansion or persistence, although superiority is not universal. | (Savoldo et al., 2011; Ramos et al., 2018) |
| Product manufacture | 7. Genetic modification | The CAR transgene is introduced into activated T cells, most commonly using replication-incompetent lentiviral or γ-retroviral vectors. Non-viral approaches, including transposon systems and messenger RNA, can also be employed. | Gene-transfer efficiency, vector copy number, genomic integration, transgene stability and potential insertional effects require evaluation. | (Hartmann et al., 2017; Ayala Ceja et al., 2024) |
| Product manufacture | 8. In-vitro expansion | Genetically modified cells are cultured with appropriate cytokines and expanded until the required therapeutic dose is achieved. | Culture duration and cytokine composition affect cellular phenotype, exhaustion, metabolic fitness and the proportions of naïve, central-memory and effector T cells. | (Levine et al., 2017; Ayala Ceja et al., 2024) |
| Product manufacture | 9. Harvesting and formulation | Expanded cells are harvested, washed and formulated in an infusion-compatible solution. Products may be administered fresh or cryopreserved for storage and transport. | Processing must preserve cell viability, sterility, potency and product identity. Cryopreservation may influence recovery and function after thawing. | (Levine et al., 2017) |
| Product manufacture | 10. Quality control and product release | The final product is tested for identity, purity, viability, sterility, endotoxin, mycoplasma, CAR expression, transduction efficiency and biological potency. | Release criteria must be predefined. Additional testing may include replication-competent virus, vector copy number and target-specific cytotoxicity. | (Levine et al., 2017; Ayala Ceja et al., 2024) |
| Clinical administration | 11. Lymphodepleting conditioning | Before infusion, patients usually receive lymphodepleting chemotherapy, commonly fludarabine and cyclophosphamide. This reduces competing lymphocytes and creates a cytokine environment favourable to CAR-T-cell expansion. | The regimen influences CAR-T-cell expansion, persistence and toxicity. The intensity may require adjustment according to disease, age and comorbidities. | (Turtle et al., 2016) |
| Clinical administration | 12. CAR-T-cell infusion | Following confirmation that the product meets release specifications, CAR-T cells are administered intravenously, usually as a single infusion but occasionally as divided or repeated doses. | Chain of identity, premedication, infusion reactions and immediate clinical observations must be carefully managed. | (Hartmann et al., 2017; Turtle et al., 2016) |
| Biological activity | 13. MHC-independent target recognition | The extracellular CAR domain binds directly to its corresponding cell-surface antigen without requiring antigen processing or presentation by the major histocompatibility complex. | CARs principally recognise surface-accessible antigens. Antigen density and accessibility influence activation and therapeutic efficacy. | (Chmielewski, Hombach and Abken, 2013) |
| Biological activity | 14. Activation and proliferation | Antigen binding induces intracellular signalling, cytokine production, metabolic activation and rapid clonal expansion of CAR-T cells. | The magnitude and duration of signalling influence efficacy, persistence, exhaustion and inflammatory toxicity. | (Kalos et al., 2011; June et al., 2018) |
| Biological activity | 15. Target-cell elimination and persistence | Activated CAR-T cells eliminate target cells through cytotoxic granule release, death-receptor signalling and cytokine-mediated mechanisms. A proportion may persist as memory cells and provide prolonged immune surveillance. | Persistent activity can support durable responses but may also cause prolonged on-target effects, such as B-cell aplasia after CD19-directed therapy. | (Kalos et al., 2011; June et al., 2018) |
| Clinical follow-up | 16. Safety and response monitoring | Patients are monitored for cytokine-release syndrome, immune effector cell-associated neurotoxicity syndrome, cytopenias, infections, organ dysfunction and treatment response. | Long-term surveillance is required to assess CAR-T-cell persistence, delayed adverse effects, immune reconstitution and disease relapse. | (June et al., 2018) |

This version is more accurate because CAR generation and MHC-independent recognition are no longer presented as manufacturing procedures. It also adds cell enrichment, activation, release testing, formulation and clinical monitoring.

Selected references

Ayala Ceja, M., Kiem, H.-P. and Wang, X. (2024) ‘CAR-T cell manufacturing: Major process parameters and next-generation strategies’, Journal of Experimental Medicine, 221(2), e20230903. https://doi.org/10.1084/jem.20230903

Chmielewski, M., Hombach, A.A. and Abken, H. (2013) ‘Antigen-specific T-cell activation independently of the MHC: chimeric antigen receptor-redirected T cells’, Frontiers in Immunology, 4, article 371. https://doi.org/10.3389/fimmu.2013.00371

Hartmann, J., Schüßler-Lenz, M., Bondanza, A. and Buchholz, C.J. (2017) ‘Clinical development of CAR T cells—challenges and opportunities in translating innovative treatment concepts’, EMBO Molecular Medicine, 9(9), pp. 1183–1197. https://doi.org/10.15252/emmm.201607485

June, C.H., O’Connor, R.S., Kawalekar, O.U., Ghassemi, S. and Milone, M.C. (2018) ‘CAR T cell immunotherapy for human cancer’, Science, 359(6382), pp. 1361–1365. [https://doi.org/10.1126/science.aar6711](https://doi.org/10.1126/science.aar6711)

Kalos, M., Levine, B.L., Porter, D.L., Katz, S., Grupp, S.A., Bagg, A. and June, C.H. (2011) ‘T cells with chimeric antigen receptors have potent antitumour effects and can establish memory in patients with advanced leukaemia’, Science Translational Medicine, 3(95), 95ra73. https://doi.org/10.1126/scitranslmed.3002842

Levine, B.L., Miskin, J., Wonnacott, K. and Keir, C. (2017) ‘Global manufacturing of CAR T cell therapy’, Molecular Therapy—Methods & Clinical Development, 4, pp. 92–101. [https://doi.org/10.1016/j.omtm.2016.12.006](https://doi.org/10.1016/j.omtm.2016.12.006)

Ramos, C.A., Rouce, R., Robertson, C.S., Reyna, A., Narala, N., Vyas, G. et al. (2018) ‘In vivo fate and activity of second- versus third-generation CD19-specific CAR-T cells in B-cell non-Hodgkin’s lymphomas’, Molecular Therapy, 26(12), pp. 2727–2737. https://doi.org/10.1016/j.ymthe.2018.09.009

Sadelain, M., Brentjens, R. and Rivière, I. (2013) ‘The basic principles of chimeric antigen receptor design’, Cancer Discovery, 3(4), pp. 388–398. [https://doi.org/10.1158/2159-8290.CD-12-0548](https://doi.org/10.1158/2159-8290.CD-12-0548)

Savoldo, B., Ramos, C.A., Liu, E., Mims, M.P., Keating, M.J., Carrum, G. et al. (2011) ‘CD28 costimulation improves expansion and persistence of chimeric antigen receptor-modified T cells in lymphoma patients’, Journal of Clinical Investigation, 121(5), pp. 1822–1826. https://doi.org/10.1172/JCI46110

Turtle, C.J., Hanafi, L.-A., Berger, C., Gooley, T.A., Cherian, S., Hudecek, M. et al. (2016) ‘CD19 CAR-T cells of defined CD4+:CD8+ composition in adult B-cell acute lymphoblastic leukaemia patients’, Journal of Clinical Investigation, 126(6), pp. 2123–2138. https://doi.org/10.1172/JCI85309
