# Supplementary material for: The future directions of CAR-T Cell therapy: unlocking the potential of immunotherapy in cancer treatment
Source: Front Mol Med. 2026 Sep 15;6:1931159. doi: 10.3389/fmmed.2026.1931159 (PMC13620351; doi:10.3389/fmmed.2026.1931159)
Supplement: Supplementary file 3 [file Supplementaryfile3.docx]

**Table 3. Selected CAR-T-Cell Therapies and Target Antigens Investigated Across Haematological and Solid Malignancies**

Developmental and regulatory status verified up to 20 August 2026.

| **CAR-T-cell therapy or platform** | **Target antigen** | **Principal cancer type** | **Developmental or regulatory status** | **Key findings and limitations** | **References** |
| --- | --- | --- | --- | --- | --- |
| **bb21217** | B-cell maturation antigen (BCMA) | Relapsed or refractory multiple myeloma | Investigational autologous CAR-T product; phase I CRB-402 study completed | Manufactured in the presence of the PI3K inhibitor bb007 to enrich for memory-like T cells. In 72 patients, the objective response rate was 69.4% and median response duration was 23.8 months. Greater early-memory composition was associated with improved expansion and response. It is distinct from the FDA-approved idecabtagene vicleucel, although both originated from the bb2121 platform. | (Alsina et al., 2026) |
| **CTL019/tisagenlecleucel (Kymriah)** | CD19 | B-cell precursor ALL, large B-cell lymphoma and follicular lymphoma | FDA approved; CTL019 was the developmental designation for tisagenlecleucel | Second-generation autologous CAR containing 4-1BB and CD3ζ signalling domains. It was the first CAR-T therapy approved by the FDA. Its principal limitations include CRS, neurological toxicity, prolonged B-cell aplasia, infections and CD19-negative relapse. | (Maude et al., 2018; Schuster et al., 2019; FDA, 2025) |
| **Axicabtagene ciloleucel (Yescarta)** | CD19 | Large B-cell lymphoma and follicular lymphoma | FDA approved | Second-generation autologous CAR containing CD28 and CD3ζ signalling domains. It produces rapid expansion and durable responses in some patients with refractory LBCL. Important limitations include CRS, ICANS, prolonged cytopenias and infection risk. | (Neelapu et al., 2017; FDA, 2026a) |
| **Brexucabtagene autoleucel (Tecartus)** | CD19 | Mantle-cell lymphoma and adult B-cell precursor ALL | FDA approved | Closely related to axicabtagene ciloleucel but manufactured using a process that includes enrichment intended to reduce malignant B-cell contamination. The ZUMA-2 study demonstrated high response rates in heavily pretreated MCL. | (Wang et al., 2020; FDA, 2026b) |
| **PSMA-directed, TGF-β-insensitive CAR-T cells** | Prostate-specific membrane antigen (PSMA) | Metastatic castration-resistant prostate cancer | Investigational; evaluated in a first-in-human phase I trial | “Armoured” CAR-T cells co-express a dominant-negative TGF-β receptor to reduce suppression by the tumour microenvironment. In the initial study, 13 patients were treated and four experienced a ≥30% reduction in prostate-specific antigen. Limited persistence, immunosuppression and treatment-associated toxicity remain obstacles. | (Narayan et al., 2022) |
| **EGFRvIII-directed CAR-T cells** | Epidermal growth factor receptor variant III (EGFRvIII) | Recurrent glioblastoma | Investigational; phase I clinical studies | EGFRvIII is tumour-specific and is not generally expressed in normal brain tissue. CAR-T cells reached glioblastoma tissue after intravenous infusion, but treatment promoted antigen loss and an immunosuppressive tumour response. A later trial combining repeated infusions with pembrolizumab did not demonstrate meaningful clinical efficacy. | (O’Rourke et al., 2017; Bagley et al., 2024) |
| **Mesothelin-directed CAR-T cells** | Mesothelin | Malignant pleural mesothelioma, ovarian cancer, lung adenocarcinoma and other mesothelin-expressing solid tumours | Investigational; several phase I programmes | Regional intrapleural administration has been explored to improve delivery to pleural tumours. In one study, mesothelin CAR-T cells followed by pembrolizumab were feasible and produced evidence of antitumour activity. A later 20-patient study found stable disease as the best response in 60%, illustrating that safety and cellular persistence have not yet translated into consistent tumour regression. | (Adusumilli et al., 2021; Barber-Rotenberg et al., 2026) |
| **MUC1- or MUC1-C-directed CAR-T cells** | Tumour-associated MUC1 epitopes, including MUC1-C, Tn-MUC1 and cleaved MUC1* | Breast, ovarian, pancreatic, colorectal, lung and other epithelial malignancies | Investigational; different autologous and allogeneic products have entered early-phase studies | MUC1 is widely expressed, but tumour-associated glycosylated or cleaved forms may provide greater specificity than total MUC1. Platforms include autologous huMNC2-CAR44/22 and allogeneic P-MUC1C-ALLO1. Major challenges are heterogeneous antigen expression, potential on-target/off-tumour toxicity, limited trafficking and poor persistence within solid tumours. | (Henry et al., 2022; Smagghe et al., 2025) |

**Abbreviations:** ALL, acute lymphoblastic leukaemia; BCMA, B-cell maturation antigen; CAR, chimeric antigen receptor; CRS, cytokine-release syndrome; EGFRvIII, epidermal growth factor receptor variant III; FDA, United States Food and Drug Administration; ICANS, immune effector cell-associated neurotoxicity syndrome; LBCL, large B-cell lymphoma; MCL, mantle-cell lymphoma; MUC1, mucin 1; PI3K, phosphoinositide 3-kinase; PSMA, prostate-specific membrane antigen; TGF-β, transforming growth factor beta.

### Summary

The table demonstrates the marked difference between the clinical success of CAR-T-cell therapy in haematological malignancies and its more limited efficacy in solid tumours. CD19-directed products such as tisagenlecleucel, axicabtagene ciloleucel and brexucabtagene autoleucel have produced durable remissions and obtained FDA approval for several B-cell malignancies. BCMA-directed CAR-T cells have similarly transformed the treatment of multiple myeloma. The investigational bb21217 product attempts to improve response durability by enriching the final product for memory-like T cells.

By comparison, CAR-T-cell therapies targeting PSMA, EGFRvIII, mesothelin and tumour-associated MUC1 remain investigational. Although early studies demonstrate that CAR-T cells can traffic to solid tumours and produce biological activity, consistent objective responses remain uncommon. Their efficacy is limited by antigen heterogeneity, antigen loss, restricted cellular trafficking, physical stromal barriers, T-cell exhaustion and the immunosuppressive tumour microenvironment. Current strategies include armoured CARs, regional administration, altered intracellular signalling, checkpoint inhibition and targeting of tumour-specific antigen isoforms.

### Harvard reference list

Adusumilli, P.S., Zauderer, M.G., Rivière, I., Solomon, S.B., Rusch, V.W., O’Cearbhaill, R.E. et al. (2021) ‘A phase I trial of regional mesothelin-targeted CAR T-cell therapy in patients with malignant pleural disease, in combination with the anti-PD-1 agent pembrolizumab’, Cancer Discovery, 11(11), pp. 2748–2763. <https://doi.org/10.1158/2159-8290.CD-21-0407>

Alsina, M., Shah, N., Jagannath, S., Kaufman, J.L., Siegel, D., Munshi, N.C. et al. (2026) ‘Anti-B-cell maturation antigen chimeric antigen receptor T-cell therapy bb21217 for relapsed and refractory multiple myeloma: results from the phase I CRB-402 study’, Cancer Immunology Research, 14(4), pp. 528–542. <https://doi.org/10.1158/2326-6066.CIR-24-0527>

Bagley, S.J., Desai, A.S., Linette, G.P., June, C.H., O’Rourke, D.M. et al. (2024) ‘Repeated peripheral infusions of anti-EGFRvIII CAR T cells in combination with pembrolizumab show no efficacy in glioblastoma: a phase I trial’, Nature Cancer. <https://doi.org/10.1038/s43018-023-00709-6>

Barber-Rotenberg, J.S., Haas, A.R., Aggarwal, C. et al. (2026) ‘Phase I study of autologous T cells bearing fully human chimeric antigen receptors targeting mesothelin in mesothelin-expressing cancers’, Molecular Therapy, 34(5). <https://doi.org/10.1016/j.ymthe.2026.01.021>

FDA (2025) ‘Kymriah (tisagenlecleucel)’. United States Food and Drug Administration. Available at: [FDA Kymriah product information](https://www.fda.gov/vaccines-blood-biologics/cellular-gene-therapy-products/kymriah) (Accessed: 20 August 2026).

FDA (2026a) ‘Yescarta (axicabtagene ciloleucel)’. United States Food and Drug Administration. Available at: [FDA Yescarta product information](https://www.fda.gov/vaccines-blood-biologics/cellular-gene-therapy-products/yescarta) (Accessed: 20 August 2026).

FDA (2026b) ‘Tecartus (brexucabtagene autoleucel)’. United States Food and Drug Administration. Available at: [FDA Tecartus product information](https://www.fda.gov/vaccines-blood-biologics/cellular-gene-therapy-products/tecartus) (Accessed: 20 August 2026).

Henry, J., Oh, D.Y., Hamilton, E.P. et al. (2022) ‘Phase I study of P-MUC1C-ALLO1 allogeneic CAR-T cells in patients with epithelial-derived cancers’, Journal for ImmunoTherapy of Cancer, 10(Suppl. 2), p. A761.

Maude, S.L., Laetsch, T.W., Buechner, J., Rives, S., Boyer, M., Bittencourt, H. et al. (2018) ‘Tisagenlecleucel in children and young adults with B-cell lymphoblastic leukaemia’, New England Journal of Medicine, 378(5), pp. 439–448. <https://doi.org/10.1056/NEJMoa1709866>

Narayan, V., Barber-Rotenberg, J.S., Jung, I.-Y., Lacey, S.F., Rech, A.J., Davis, M.M. et al. (2022) ‘PSMA-targeting TGF-β-insensitive armoured CAR T cells in metastatic castration-resistant prostate cancer: a phase I trial’, Nature Medicine, 28(4), pp. 724–734. <https://doi.org/10.1038/s41591-022-01726-1>

Neelapu, S.S., Locke, F.L., Bartlett, N.L., Lekakis, L.J., Miklos, D.B., Jacobson, C.A. et al. (2017) ‘Axicabtagene ciloleucel CAR T-cell therapy in refractory large B-cell lymphoma’, New England Journal of Medicine, 377(26), pp. 2531–2544. <https://doi.org/10.1056/NEJMoa1707447>

O’Rourke, D.M., Nasrallah, M.P., Desai, A., Melenhorst, J.J., Mansfield, K., Morrissette, J.J.D. et al. (2017) ‘A single dose of peripherally infused EGFRvIII-directed CAR T cells mediates antigen loss and induces adaptive resistance in patients with recurrent glioblastoma’, Science Translational Medicine, 9(399), eaaa0984. <https://doi.org/10.1126/scitranslmed.aaa0984>

Schuster, S.J., Bishop, M.R., Tam, C.S., Waller, E.K., Borchmann, P., McGuirk, J.P. et al. (2019) ‘Tisagenlecleucel in adult relapsed or refractory diffuse large B-cell lymphoma’, New England Journal of Medicine, 380(1), pp. 45–56. <https://doi.org/10.1056/NEJMoa1804980>

Smagghe, B.J., Bamdad, C.C. et al. (2025) ‘Effective CAR T-cell targeting of an MUC1 cleavage product’, Journal for ImmunoTherapy of Cancer, 13, e010342.

Wang, M., Munoz, J., Goy, A., Locke, F.L., Jacobson, C.A., Hill, B.T. et al. (2020) ‘KTE-X19 CAR T-cell therapy in relapsed or refractory mantle-cell lymphoma’, New England Journal of Medicine, 382(14), pp. 1331–1342. <https://doi.org/10.1056/NEJMoa1914347>
